# Anti‐Inflammatory Macrophage‐Derived Exosomes Modified With Self‐Antigen Peptides for Treatment of Experimental Autoimmune Encephalomyelitis

**DOI:** 10.1002/advs.202415265

**Published:** 2025-02-12

**Authors:** Qiaoyun Li, Jinwon Park, Jung Suk Kim, Quoc‐Viet Le, Jaiwoo Lee, Yu‐Kyoung Oh

**Affiliations:** ^1^ College of Pharmacy and Research Institute of Pharmaceutical Sciences Seoul National University Seoul 08826 Republic of Korea; ^2^ Faculty of Pharmacy Ton Duc Thang University Ho Chi Minh City 700000 Vietnam; ^3^ College of Pharmacy Korea University Sejong 30019 Republic of Korea

**Keywords:** anti‐inflammatory macrophage‐derived exosome, autoimmune disease, encephalomyelitis, immune tolerance, self‐antigen modification

## Abstract

Current treatments for autoimmune diseases often involve broad‐acting immunosuppressants, which carry risks such as infections and malignancies. This study investigates whether exosomes derived from anti‐inflammatory macrophages (AE) and decorated with myelin oligodendrocyte glycoprotein (MOG) peptide (AE/M) can induce immune tolerance in autoimmune diseases. Experimental autoimmune encephalomyelitis (EAE), a mouse model for multiple sclerosis, serves as the autoimmune disease model. Exosomes derived from myoblasts or fibroblasts are also modified with MOG peptides for comparison. Unlike their myoblast or fibroblast counterparts, exosomes from anti‐inflammatory macrophages demonstrate a targeted capacity toward antigen‐presenting cells. Moreover, AE/M uniquely promotes the differentiation of dendritic cells (DC) into a tolerogenic phenotype. When splenocytes are treated with AE/M, an increased population of tolerogenic DC (tolDC) is observed, even under proinflammatory stimuli. Subcutaneous administration of AE/M in the EAE mouse model results in MOG peptide‐specific immune tolerance and preserves motor coordination. In contrast to treatments with fibroblast‐ or myoblast‐derived exosomes modified with MOG peptides, AE/M treatment provides complete protection from EAE in mice. These findings highlight the potential of self‐antigen modified AE as a versatile and adaptable nanoplatform for the treatment of various autoimmune diseases.

## Graphical abstract

Anti‐inflammatory macrophage‐derived exosomes modified with selfantigen peptides are developed to induce antigen‐specific immune tolerance. These exosomes demonstrate prophylactic effects in experimental autoimmune encephalomyelitis.

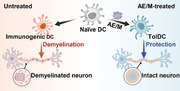

## 1 Introduction

Autoimmune diseases, marked by abnormal immune responses against self‐antigens, lead to self‐tissue or organ damage.^[^ ^1^ ^]^ The suppression of hyperactive immune cells is widely recognized as an effective strategy for these conditions. Nanomaterials that modulate immune cell activity have become increasingly prominent in autoimmune disease treatment. Synthetic nanomaterials, including cerium oxide nanoparticles and carbon‐based materials, offer unique mechanisms in the treatment of autoimmune diseases. Cerium oxide nanoparticles, known for their reactive oxygen species (ROS) scavenging activity, have shown effectiveness in the experimental autoimmune encephalomyelitis mouse model.^[^ ^2^ ^]^ Additionally, graphene oxide nanosheets are effective in modulating neuroinflammation, chiefly by diminishing Ca^2+^ signaling in astrocytes, thereby contributing to multiple sclerosis therapy.^[^ ^3^ ^]^ Furthermore, lipid nanoparticles have been employed to deliver nucleic acids, such as tumor necrosis factor (TNF)‐*α* siRNA, offering a novel treatment approach for autoimmune diseases, including rheumatoid arthritis.^[^ ^4^ ^]^

Current research on nanomaterials for autoimmune disease treatment, including their role as drug delivery systems, faces several challenges. A primary concern is that immune‐regulating agents or nanomaterials can induce systemic immunosuppression, potentially leading to opportunistic infections and toxicity. For instance, dexamethasone, commonly carried in nanomaterials for autoimmune diseases, is an effective glucocorticoid but may increase the risk of osteoporosis or metabolic disorders.^[^ ^5^ ^]^ Therefore, targeting specific immune responses against self‐antigens is crucial.

Biodistribution poses another significant challenge, particularly in diseases like multiple sclerosis, where the central nervous system (CNS) is the primary site of pathology and is protected by the blood–brain barrier (BBB). Functional nanomaterials need to effectively cross these barriers to yield successful outcomes.^[^ ^6^ ^]^ For conditions like multiple sclerosis, it is essential that nanomaterials are effectively distributed to the CNS, traversing the BBB to provide effective treatment.

Tolerogenic dendritic cells (tolDC) are integral to the peripheral tolerance system, regulating a range of immune cells, including T cells, B cells, macrophages, and dendritic cells (DC) themselves. They play a crucial role in inducing clonal deletion or anergy in T or B cells that mistakenly recognize self‐antigens as harmful. This function of tolDC is essential in eliminating autoreactive leukocytes, forming a core component of autoimmune disease therapy.^[^ ^7^ ^]^ Notably, the therapy involving tolDC does not necessitate BBB penetration by nanomaterials due to the role of T cells.

Exosomes are extracellular vesicles originating from endosomal compartments and are secreted by various cell types for intercellular communication. Given their potent biological activity and diverse cargo, exosomes have been extensively explored as drug delivery systems. In medical applications involving exosomes, the choice of source cell is critical as it imparts similar properties to the exosomes, owing to their analogous cargo.^[^ ^8^, ^9^ ^]^

Macrophages, as key leukocytes in the innate immune system, play dual roles in host defense and physiological homeostasis. Reflecting this diversity, exosomes derived from macrophages also exhibit a range of biological effects.^[^ ^10^ ^]^ Macrophages are typically categorized into proinflammatory and anti‐inflammatory types. Proinflammatory macrophages, known as classically activated macrophages, are characterized by their immune stimulation activity. In contrast, anti‐inflammatory macrophages primarily exhibit immune suppressive functions, contributing to tissue repair and immune response regulation.^[^ ^11^ ^]^ Consequently, utilizing exosomes derived from anti‐inflammatory macrophages holds promise for autoimmune disease therapy, such as in rheumatoid arthritis.^[^ ^12^ ^]^

**Figure 1:**
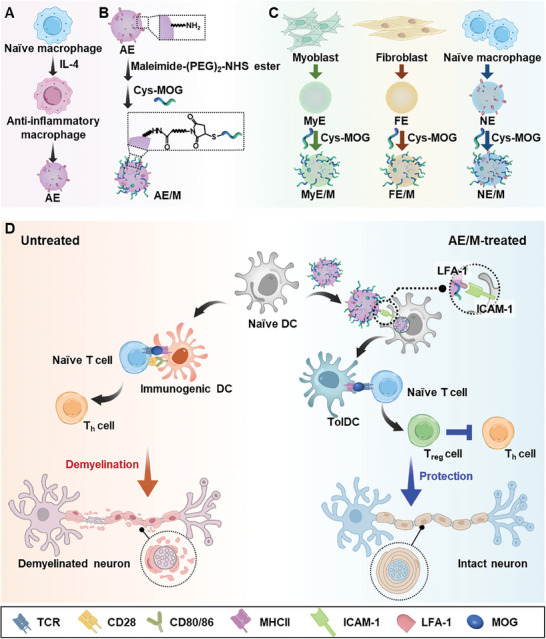
Proposed mechanism of AE/M to induce antigen‐specific immune tolerance. A) Anti‐inflammatory macrophages were generated from naïve macrophages using interleukin 4 (IL‐4) stimulation. Subsequently, anti‐inflammatory macrophage‐derived exosomes were isolated from these anti‐inflammatory macrophages. B) MOG peptide, serving as a model self‐antigen, was conjugated to the surface of AE using maleimide‐(PEG)_2_‐NHS ester. Cysteine‐modified MOG peptide (Cys‐MOG) was conjugated to the exosomes via the thiol group and maleimide moiety, resulting in the formation of AE/M. C) Exosomes were also isolated from myoblasts (MyE), fibroblasts (FE), and naïve macrophages (NE). Following MOG peptide conjugation, these exosomes were termed MyE/M, FE/M, and NE/M, respectively. D) A schematic illustration outlines the proposed mechanism of action for AE/M in protecting mice from EAE. The treatment with AE/M is proposed to induce MOG‐presenting tolDC. The tolDC migrated to lymph nodes and facilitated the generation of T_reg_ cells, which play a key role in suppressing autoimmune activation and protecting neurons.

This study explores the hypothesis that exosomes derived from anti‐inflammatory macrophages (AE) may retain the immunosuppressive functions of anti‐inflammatory macrophages and that their modification with self‐antigens could induce antigen‐specific immune tolerance. The results demonstrate that AE, when modified with myelin oligodendrocyte glycoprotein (MOG) peptide (AE/M), can confer antigen‐specific immune tolerance and promote regulatory T cells (T_reg_), thereby protecting mice from inflammatory symptoms in the experimental autoimmune encephalomyelitis model (**Figure** **1**).

## 2 Results

### 2.1 Characterization of Exosomes

**Figure 2:**
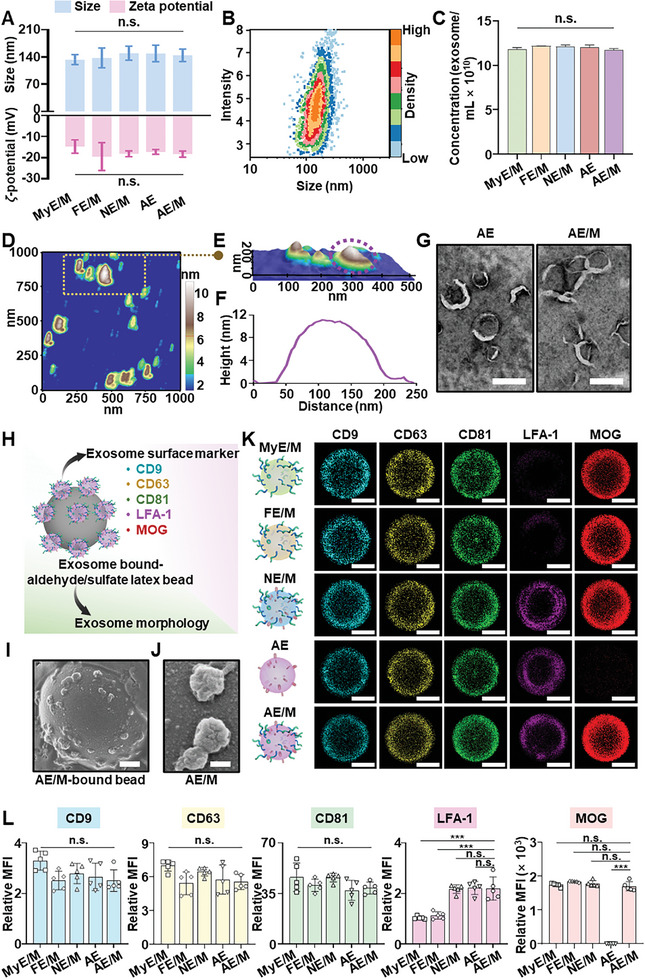
Characterization of MOG‐conjugated exosomes. A) Size and zeta potential of different exosomes (*n* = 5). B) Size distribution of AE/M obtained by nanoparticle tracking analysis. C) Concentration of exosomes counted through nanoparticle tracking analysis (*n* = 5). D) 2D AFM image of AE/M. E) 3D AFM image of AE/M. F) The height and length data for the AE/M highlighted in (E). G) Morphology of AE and AE/M was visualized using transmission electron microscopy (TEM). Scale bar: 200 nm. H) A schematic illustration of AE/M‐bound aldehyde/sulfate latex beads which was used for exosome visualization and analysis of exosome surface markers. I) Observation of AE/M‐bound aldehyde/sulfate latex beads by scanning electron microscopy (SEM). AE/M was allowed to be adsorbed on aldehyde/sulfate latex beads followed by SEM observation. Scale bar: 500 nm. J) SEM image of AE/M that was adsorbed on aldehyde/sulfate latex beads. Scale bar: 100 nm. K) Visualization of exosome surface markers by confocal microscopy. Scale bar: 2 µm. Anti‐CD9 antibody, anti‐CD63 antibody, anti‐CD81 antibody, and anti‐LFA‐1 antibody were used to stain exosomes after the adsorption of exosomes onto aldehyde/sulfate latex beads. Primary anti‐MOG antibody was used to stain MOG peptides followed by the second antibody staining for the detection. L) Relative mean fluorescence intensity of exosome surface markers obtained by flow cytometry (*n* = 5). (Data are presented as the mean ± standard deviation (SD). n.s., not significant; ***p < 0.001).

Physical properties of various exosomes were characterized in terms of size, zeta potential, morphology, and the number of exosomes per unit volume. Regardless of origin cells and surface modification, the sizes of exosomes did not significantly differ, showing average sizes in the range of 133 ± 13 to 150 ± 18 nm (**Figures** **2**A,B and S1, Supporting Information). The conjugation of MOG peptides did not alter the surface charge of the exosomes. Zeta potential value of AE/M was −18 ± 1.5 mV, while those of MyE/M and AE were −15 ± 3.1 and −17 ± 1.1 mV, respectively (Figure 2A). Nanoparticle tracking analysis revealed that the concentration of AE/M suspension was 1.2 × 10^11^ exosome mL^−1^, and no significant difference existed among all groups (Figure 2C). In addition, the representative nanoparticle tracking analysis of AE/M recorded Brownian motion (Video S1, Supporting Information). AE/M exhibited a round shape when analyzed under atomic force microscopy (AFM) (Figure 2D–F), and the same morphology was also observed in other exosomes (Figure S2, Supporting Information). Consistent with the AFM data, the morphology of AE and AE/M showed a similar appearance, indicating that peptide conjugation did not affect the shape of the exosomes (Figure 2G).

The MOG peptide was conjugated to the surface of exosomes derived from myoblasts, fibroblasts, naïve macrophages, and anti‐inflammatory macrophages using an NHS‐maleimide linker (Figure S3B–E, Supporting Information). Aldehyde/sulfate latex beads were coated with exosomes (Figure 2H), and the morphology of beads bound with exosomes was observed (Figure 2I,J). Using the beads bound with exosomes, various surface markers, including CD9, CD63, CD81,^[^ ^13^ ^]^ and conjugated MOG were visualized via confocal microscopy (Figure 2K), and fluorescence intensity was assessed by flow cytometry (Figure 2L). CD9, CD63, and CD81, as common surface markers, were uniformly detectable across all exosome types, with no significant differences in expression levels (Figure 2K,L). Lymphocyte function‐associated antigen 1 (LFA‐1) only could be found in macrophage‐derived exosomes (Figure 2K,L).

The fluorescence from conjugated‐MOG was observed in all groups except plain AE indicating successful conjugation of MOG peptide to various exosomes (Figure 2K). The fluorescence intensity of LFA‐1 was 2.1 times and 1.9 times higher in AE/M than in MyE/M and FE/M, respectively (Figure 2L). The fluorescence intensity of MOG was significantly higher in AE/M compared to AE. Additionally, no significant difference existed in MOG‐conjugation among MyE/M, FE/M, NE/M, and AE/M (Figure 2L). Consistent with the flow cytometry data, the quantified confocal images of CD9, CD63, and CD81 showed no statistically significant differences. The mean fluorescence intensity of MOG in AE showed almost no signals. In contrast, the mean fluorescence intensity of LFA‐1 in macrophage‐derived exosomes was significantly higher than that in the MyE/M and FE/M (Figure S4, Supporting Information).

The amount of conjugated MOG peptide was determined by calculating the difference between the total and unconjugated MOG peptides, which were quantified using high‐performance liquid chromatography (HPLC) analysis (Figure S5A, Supporting Information). The free MOG peptide showed a retention time of 5.8 min, which was also observed in the AE/M group sample. In contrast, the AE group sample did not exhibit a peak at 5.8 min, indicating the absence of MOG peptides (Figure S5B, Supporting Information). The amount of unconjugated MOG peptide was quantified using a standard curve (Figure S5C, Supporting Information). HPLC analysis revealed that 82.6% of the added MOG peptide was conjugated to the surface of AE, resulting in a final concentration of 66.1 µg of MOG per 1.2 × 10¹¹ AE/M. Similar conjugation efficiency and MOG concentrations were observed across all groups (Figure S5D,E, Supporting Information).

### 2.2 Induction of TolDC

**Figure 3:**
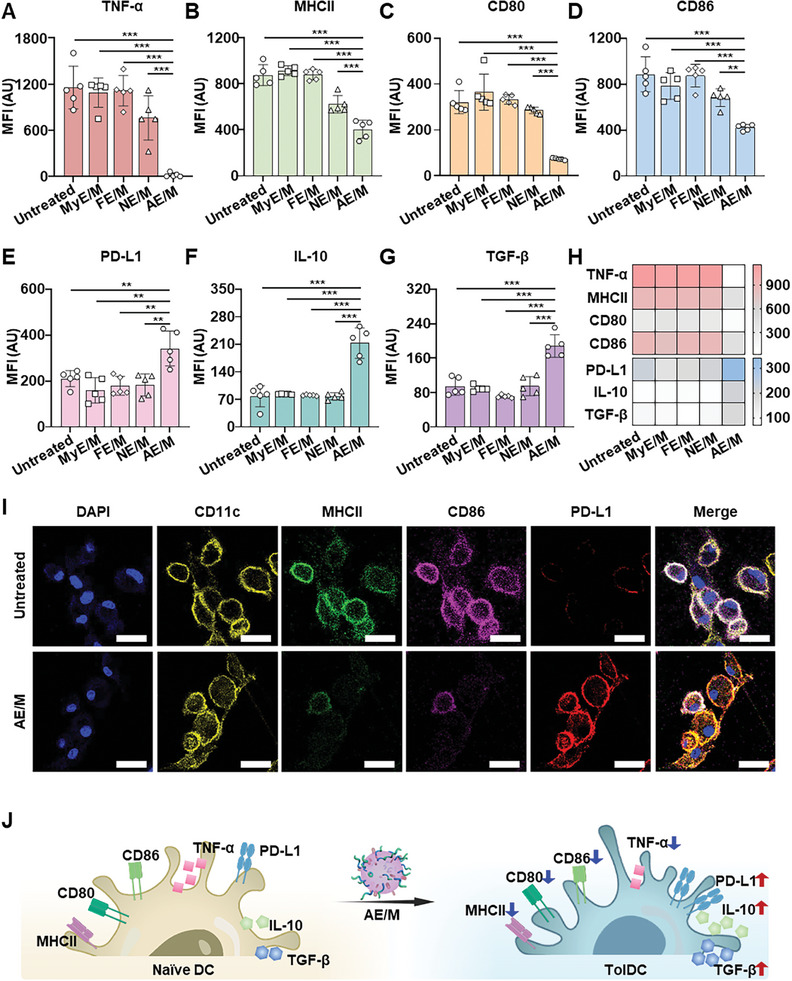
Induction of tolDC by AE/M. A–D) The mean fluorescence intensity of proinflammatory markers, including TNF‐*α* A), MHCII B), CD80 C), and CD86 D) was measured (*n* = 5). E–G) The mean fluorescence intensity of anti‐inflammatory markers, including PD‐L1 E), IL‐10 F), and TGF‐*β* G) was measured (*n* = 5). H) Summary of the expression of inflammation‐relevant markers after treatment. I) Visualization of the expression of MHCII, CD86, and PD‐L1 in DC after AE/M treatment via confocal microscopy. Scale bar: 20 µm. J) A schematic illustration showing that AE/M treatment induced tolDC as manifested by the increase in anti‐inflammatory markers and the decrease in proinflammatory markers. (Data are presented as the mean ± SD. **p < 0.01; ***p < 0.001).

The efficacy of various exosome formulations in inducing tolDC was assessed by evaluating the expression of inflammation markers. Flow cytometry analysis revealed that proinflammatory markers, including TNF‐*α*, major histocompatibility complex class II molecule (MHCII), CD80, CD86, and interleukin 6 (IL‐6), had significantly lower expression levels in the AE/M‐treated group compared to other groups (**Figures** **3**A–D and S6A, Supporting Information). Specifically, the expression levels of TNF‐*α*, MHCII, CD80, CD86, and IL‐6 in the AE/M‐treated group were reduced by 35.5‐fold, 1.5‐fold, 3.9‐fold, 1.6‐fold, and 2.0‐fold, respectively, relative to the NE/M‐treated group. In contrast, AE/M treatment significantly upregulated the expression of anti‐inflammatory markers, including programmed death ligand‐1 (PD‐L1), interleukin 10 (IL‐10), and transforming growth factor (TGF)‐*β* (Figure 3E–G). Compared to the NE/M‐treated group, the AE/M‐treated group exhibited increased expression of PD‐L1 by 1.9‐fold, IL‐10 by 2.7‐fold, and TGF‐*β* by 2.0‐fold.

Fluorescent intensity comparisons of anti‐inflammatory and proinflammatory markers for each treatment group are presented (Figure 3H). No significant difference was observed in the expression levels of inflammation markers, such as MHCII, CD86, CD80, and PD‐L1 between AE and AE/M, suggesting that the modification with MOG did not alter the biological effect of AE (Figure S6A–H, Supporting Information). Confocal microscopy of DC indicated a lower fluorescent intensity of MHCII, CD86, and CD80, but a higher intensity of PD‐L1 in the AE/M‐treated group compared to the untreated group (Figures 3I and S6I, Supporting Information). An overview illustrating the increase in anti‐inflammatory markers and the decrease in proinflammatory markers due to AE/M treatment is provided (Figure 3J).

The effects of various exosomes on bone marrow‐derived dendritic cells (BMDC) and bone marrow‐derived macrophages (BMDM) were evaluated by measuring specific inflammation‐related markers. In BMDC, AE/M reduced CD86 expression by 1.8‐fold compared to NE/M, with no significant difference between AE and AE/M (Figure S7A,B, Supporting Information). The TNF‐*α* level in the BMDC culture medium of the AE/M‐treated group decreased by 1.5‐fold compared to the NE/M‐treated group (Figure S7C, Supporting Information).

Similarly, AE/M alleviated inflammation in BMDM, as demonstrated by reductions in CD86 expression, intracellular ROS, and TNF‐*α* secretion. Specifically, CD86 expression in the AE/M‐treated group decreased by 1.3‐fold compared to the NE/M‐treated group (Figure S7D,E, Supporting Information). AE/M treatment also reduced intracellular ROS levels in BMDM, as indicated by a 1.7‐fold decrease in CM‐H2DCFDA intensity compared to NE/M treatment (Figure S7F,G, Supporting Information). Finally, TNF‐*α* secretion by BMDM in the AE/M‐treated group was reduced by 1.4‐fold relative to the NE/M‐treated group (Figure S7H, Supporting Information).

### 2.3 Uptake of Exosomes by Antigen‐Presenting Cells

**Figure 4:**
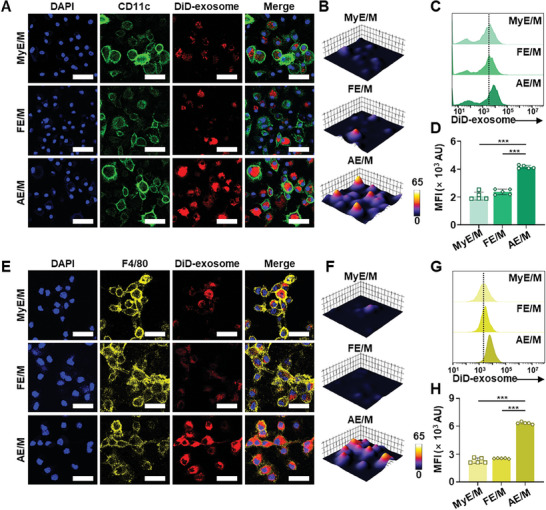
Uptake of MOG‐modified exosomes by DC and macrophages. A) Cellular uptake of MOG‐modified exosomes by DC was visualized by confocal microscopy. Scale bar: 40 µm. B) 3D surface plot of DiD‐exosome obtained from confocal images shown in (A). C) Histogram of cellular uptake of MOG‐modified exosomes by DC was assessed by flow cytometry. D) Mean fluorescence intensity of DC uptake of MOG‐modified exosomes (*n* = 5). E) Cellular uptake of MOG‐modified exosomes by macrophages was visualized by confocal microscopy. Scale bar: 20 µm. F) 3D surface plot of DiD‐exosome obtained from confocal images shown in (E). G) Histogram of macrophage uptake of MOG‐modified exosomes was determined by flow cytometry. H) Mean fluorescence intensity of macrophage uptake of MOG‐modified exosomes (*n* = 5). (Data are presented as the mean ± SD. ***p < 0.001).

The cellular uptake of exosomes by antigen‐presenting cells, specifically DC and macrophages, varied based on the cell origin from which the exosomes were derived. Confocal microscopy analysis indicated that AE/M was taken up by DC to a greater extent than MyE/M and FE/M (**Figure** **4**A,B). Quantitative flow cytometry results showed that DC uptake of AE/M was 2.0‐fold and 1.7‐fold higher than that of MyE/M and FE/M, respectively (Figure 4C,D). Additionally, confocal microscopy revealed an increasing uptake of AE/M by DC over time (Figure S8, Supporting Information).

In a similar pattern to DC, macrophages exhibited a higher uptake of AE/M compared to MyE/M and FE/M (Figure 4E,F). The macrophage uptake of AE/M was 2.7 times and 2.5 times greater than that of MyE/M and FE/M, respectively (Figure 4G,H). It is notable that the uptake of AE/M by DC and macrophages did not show a significant difference when compared with the uptake of AE and NE/M (Figure S9, Supporting Information).

The effect of macrophage exosomes on targeting antigen‐presenting cells was further evaluated using splenocytes isolated from EAE mice. Flow cytometry analysis showed that AE/M uptake by DC was 2.7‐fold and 2.9‐fold higher compared to MyE/M and FE/M, respectively (Figure S10A, Supporting Information). AE/M showed significantly enhanced targeting of macrophages, with a 2.0‐fold increase in macrophage uptake compared to MyE/M and FE/M (Figure S10B, Supporting Information).

### 2.4 Biodistribution of Exosomes

**Figure 5:**
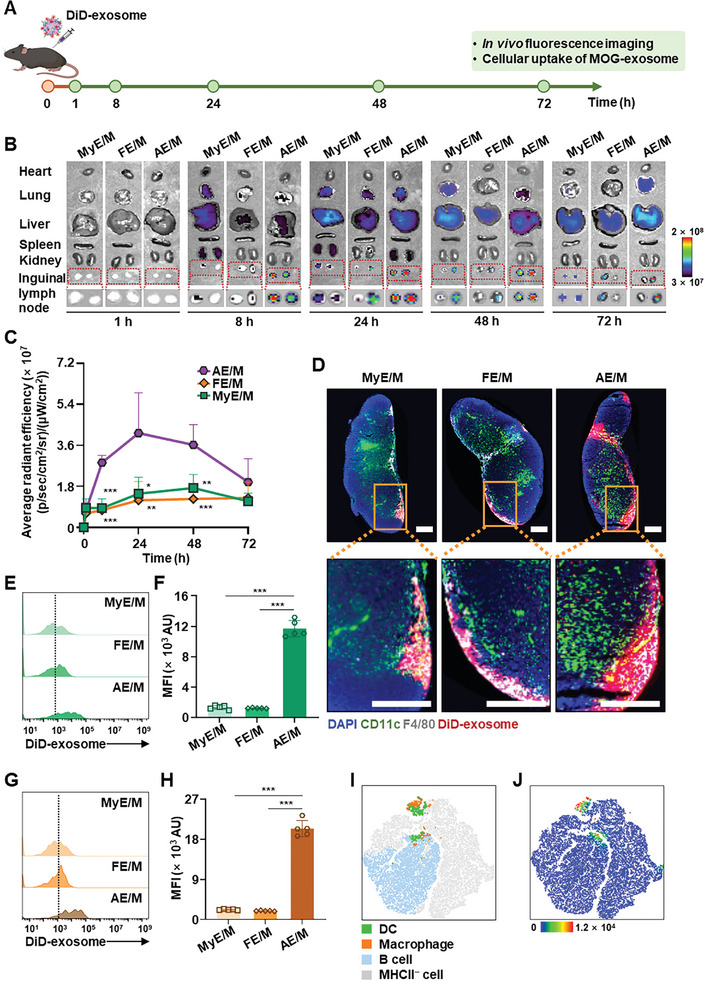
Biodistribution of MOG‐conjugated exosomes. A) The schedule of the biodistribution study is outlined. DiD‐labeled MOG‐conjugated exosomes were administered subcutaneously. At different time points, in vivo fluorescence imaging was performed using the IVIS instrument. Lymph nodes were harvested for further cellular analysis. B) Images show the biodistribution of different MOG‐modified exosomes as determined by the IVIS instrument. C) The fluorescence intensity of exosomes in inguinal lymph nodes was measured at different time points (*n* = 5). D) Immunofluorescence images show the accumulation of MOG‐modified exosomes in lymph nodes 24 h after administration. Tissue sections were prepared, and immunostaining was conducted using 4′,6‐diamidino‐2‐phenylindole (DAPI) (blue), anti‐CD11c antibody (green), and anti‐F4/80 antibody (gray). The scale bars indicate 0.25 mm for the upper and lower images. E,F) Cellular uptake of MOG‐modified exosomes by DC in lymph nodes: histogram plot E), and mean fluorescence intensity F). DiD‐labeled MOG‐modified exosomes were subcutaneously injected followed by collection of lymph nodes at 24 h. DiD^+^ immune cells were analyzed by flow cytometry (*n* = 5). G,H) Cellular uptake of MOG‐modified exosomes by macrophages: histogram plot G), and mean fluorescence intensity H) (*n* = 5). I) *t*‐SNE visualization of different immune cell populations from lymph nodes isolated from AE/M‐treated mice. J) *t*‐SNE heatmap statistic of DiD^+^ cells in lymph nodes from AE/M‐treated mice. (Data are presented as the mean ± SD. *p < 0.05; **p < 0.01; ***p < 0.001).

To evaluate the in vivo fate of exosomes, DiD‐labeled MOG‐conjugated exosomes were administered subcutaneously to mice (**Figure** **5**A). The AE/M group showed a higher distribution to the lymph nodes compared to the MyE/M and FE/M groups from 8 h post‐dose (Figure 5B). At 24 h postadministration, the fluorescence intensity in AE/M‐treated mice was 2.8 and 3.5 times greater than that in MyE/M‐ and FE/M‐treated mice, respectively, marking the peak fluorescence intensity. However, at 48 and 72 h postinjection, the signals decreased, indicating exosome excretion from the lymph nodes (Figure 5C).

Fluorescence imaging of lymph node tissue sections showed a higher distribution of AE/M in lymph nodes and notable colocalization with DC and macrophages (Figure 5D). Flow cytometry was used to quantify the presence of DiD^+^ CD11c^+^ DC and DiD^+^ F4/80^+^ macrophages. AE/M showed significantly greater colocalization with CD11c^+^ DC compared to MyE/M and FE/M (Figure 5E), with fluorescence intensity exceeding 9‐fold (Figure 5F). Similarly, AE/M was more readily taken up by macrophages in lymph node tissues (Figure 5G). The DiD intensity in the AE/M‐treated group was 9.2‐fold and 10‐fold higher than that in the MyE/M and FE/M‐treated groups, respectively (Figure 5H). Further analysis of DiD^+^ cell profiles in lymph nodes indicated that AE/M predominantly colocalized with DC and macrophages (Figure 5I,J). Moreover, AE/M showed a comparable distribution and uptake by antigen‐presenting cells when compared with AE and NE/M (Figure S11, Supporting Information).

### 2.5 Prophylactic Effect of AE/M on EAE Mice

**Figure 6:**
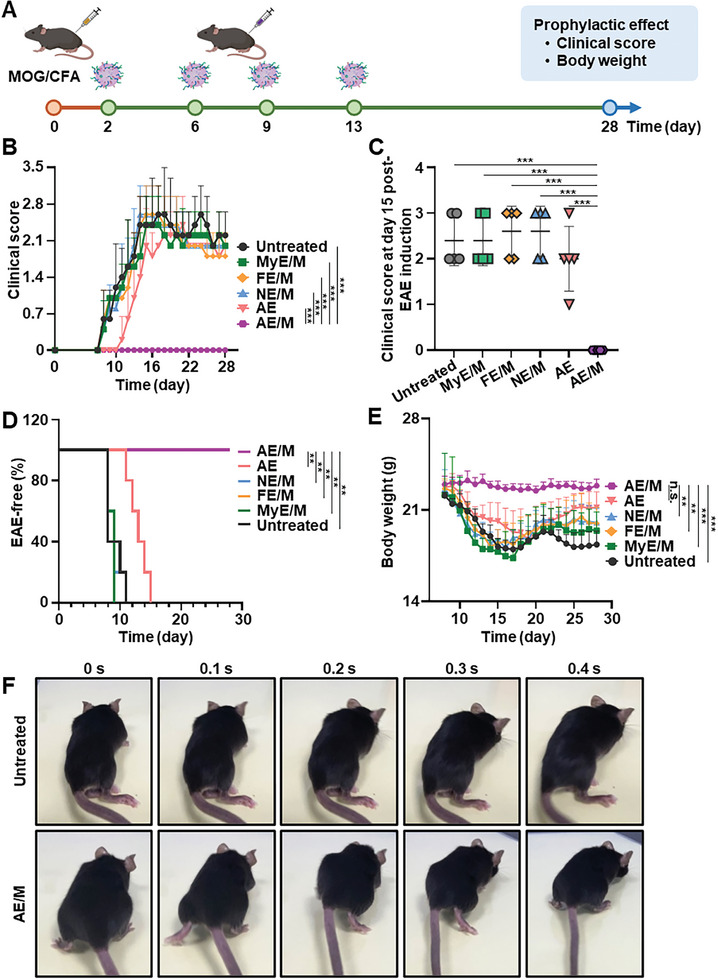
In vivo prophylactic effect of AE/M on EAE mice. A) A schematic illustration of the schedule of in vivo study. Two days after EAE model induction (prior to EAE onset), various exosome formulations were administered through subcutaneous injection on days 2, 6, 9, and 13. Clinical score and body weight were recorded. B) Clinical score of the mice treated with different MOG‐conjugated exosomes (*n* = 5). C) Clinical score at day 15 post‐EAE induction (*n* = 5). D) The percentage of EAE‐free mice after immunization in each group (*n* = 5). E) Body weight of the mice treated with different exosome formulations (*n* = 5). F) Photographs of the mice from the untreated group and the AE/M‐treated group showing walking behavior. Live movies are provided in Video S2 (Supporting Information) (Data are presented as the mean ± SD. n.s., not significant; **p < 0.01; ***p < 0.001).

To investigate prophylactic effects of AE/M, various exosome formulations were subcutaneously administered to mice prior to EAE onset (on day 2 post‐EAE induction), with intervals of 3 or 4 days between each dose, totaling four administrations (**Figure** **6**A). The MyE/M, FE/M, and NE/M‐treated groups began exhibiting disease symptoms on day 8, peaking on day 15, like the disease progression of the untreated group (Figure 6B,C). In contrast, AE/M treatment prevented the onset of symptoms, maintaining a consistent symptom score of 0.0 throughout the experimental period (Figure 6B,D).

The incidence of EAE varied among the groups treated with different MOG‐modified exosomes. All groups, except the AE/M group, showed symptoms of EAE (Figure 6D). The group treated with AE exhibited a slower progression of EAE compared to the MyE/M and FE/M groups but did not fully prevent EAE onset. In comparison, AE/M administration offered complete protection against EAE induction. Furthermore, only mice in the AE/M‐treated group maintained their body weight throughout the in vivo experiment. In contrast, the body weight of mice in all other groups consistently decreased (Figure 6E). Mice in the untreated group displayed severe motor impairments, including limp tail and complete paralysis of the hind legs or on one side of the body (Figure 6F; and Video S2A, Supporting Information). Conversely, mice treated with AE/M maintained favorable motor coordination (Figure 6F; and Video S2B, Supporting Information).

Additionally, AE/M was intravenously administered prior to EAE onset (on day 2 post‐EAE induction) (Figure S12A, Supporting Information). The group receiving intravenous AE/M treatment exhibited better prophylactic effect compared to the untreated and AE‐treated groups, as evidenced by its significantly lower clinical scores (Figure S12B, Supporting Information). The body weights of mice treated with intravenous AE/M were higher than those of mice that were untreated or treated with intravenous AE (Figure S12C, Supporting Information).

The prophylactic effect of AE/M was further investigated by administering the intervention on the day of EAE onset. AE/M was subcutaneously administered on day 9 after EAE induction, with intervals of 3 or 4 days between each dose, totaling four administrations (Figure S13A, Supporting Information). Among the groups tested, AE/M showed the highest prophylactic benefits for EAE. The AE/M treatment significantly reduced symptom severity compared to AE treatment alone (Figure S13B, Supporting Information). The maximum clinical score in the AE/M‐treated group was 1.6‐fold lower than that in the AE‐treated group (Figure S13B, Supporting Information). The body weights of mice treated with AE/M were higher than those of mice that were treated with AE (Figure S13C, Supporting Information).

### 2.6 Immunomodulation by Subcutaneous Administration of AE/M

**Figure 7:**
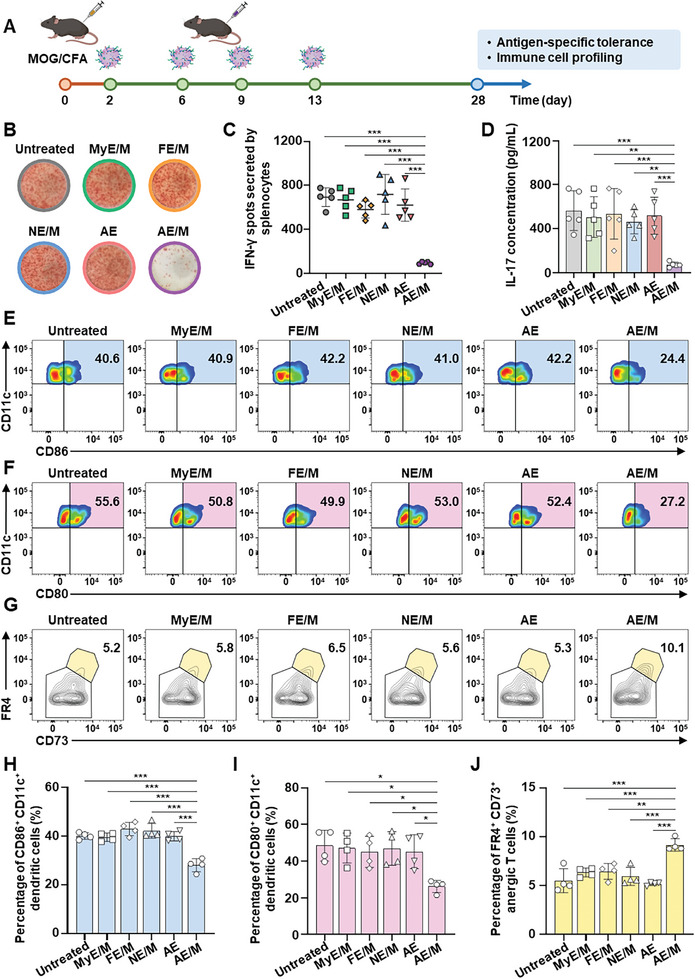
Induction of antigen‐specific immune tolerance by subcutaneous administration of exosomes. A) A schematic illustration of the in vivo study. Subcutaneous administration of MOG‐conjugated exosomes started 2 days after EAE model induction. The administration interval was 3 or 4 days. 4 weeks after immunization, spleens were harvested for further analysis. B) Photographs of red spots generated by MOG‐responsive splenocytes from mice treated with different formulations. 28 days after EAE model induction, spleens were harvested, and splenocytes were stimulated in vitro with 5 µg mL^−1^ of MOG_35–55_ for 24 h followed by IFN‐*γ* enzyme‐linked immunosorbent spot (ELISpot) assay. C) The number of IFN‐*γ* spots generated by MOG‐responsive splenocytes from mice treated with various exosomes (*n* = 5). D) The concentration of IL‐17 in the medium collected from splenocytes restimulated with 10 µg mL^−1^ of MOG_35–55_ (*n* = 5). E–J) Immunophenotyping of spleens was performed using flow cytometry. E) Plots show CD86^+^ CD11c^+^ DC in the spleens. F) Plots show CD80^+^ CD11c^+^ DC in the spleens. G) Representative plots show the population of anergic T cells in the spleens following exosome treatment. H) The frequency of CD86^+^ CD11c^+^ DC in the spleens was measured (*n* = 4). I) The frequency of CD80^+^ CD11c^+^ DC in the spleens was measured (*n* = 4). J) The percentage of anergic T cells in the spleens was measured (*n* = 4). (Data are presented as the mean ± SD. *p < 0.05; **p < 0.01; ***p < 0.001).

To assess the impact of AE/M on immunomodulation, the levels of interferon‐*γ* (IFN‐*γ*) and interleukin 17 (IL‐17) secreted by MOG_35–55_‐restimulated splenocytes were quantified. Furthermore, the assessment of immune tolerance involved analyzing immune cell populations in the spleens via flow cytometry, following subcutaneous administration of various MOG‐conjugated exosomes (**Figure** **7**A). The AE/M treatment significantly decreased immune responses against the self‐antigen MOG_35–55_, indicating the establishment of MOG‐specific immune tolerance. This was demonstrated by the significantly lower levels of IFN‐*γ* and IL‐17 in the AE/M‐treated group compared to other groups. In the AE/M‐treated group, the count of IFN‐*γ* spots was 6.6 times lower than that in the AE‐treated group (Figure 7B,C). Additionally, the AE/M treatment resulted in a 7.2‐fold reduction in IL‐17 concentration relative to the AE treatment (Figure 7D).

Immunophenotyping revealed that AE/M treatment induced peripheral tolerance, as indicated by a decrease in activated DC and an increase in anergic T cells in the spleens. AE/M treatment significantly reduced the expression of CD86 and CD80 in DC compared to AE treatment. The population of CD86^+^ DC in the AE/M‐treated group was 1.4‐fold lower than that in the AE‐treated group (Figure 7E,H). Similarly, AE/M treatment decreased the population of CD80^+^ DC by 1.7‐fold compared to AE treatment (Figure 7F,I). The population of anergic T cells increased by 1.8‐fold in the AE/M‐treated group compared to the AE‐treated group (Figure 7G,J).

### 2.7 Immune Microenvironment of CNS Following Exosome Treatments

**Figure 8:**
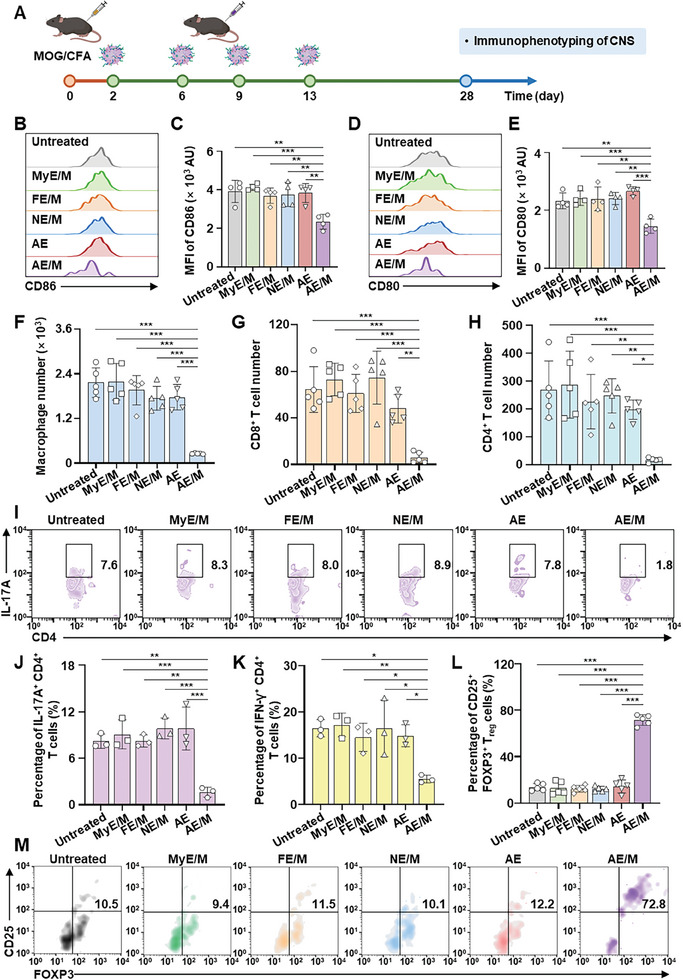
Immune microenvironment of CNS following exosome treatments. A) A schematic illustration of the in vivo study. Subcutaneous administration of MOG‐conjugated exosomes started 2 days after EAE model induction. The administration interval was 3 or 4 days. 4 weeks after immunization, spinal cords were harvested for further analysis. B) Representative flow cytometry plots show the level of CD86 on DC. C) The mean fluorescence intensity of CD86 on DC was measured (*n* = 4). D) Representative flow cytometry plots show the level of CD80 on DC. E) The mean fluorescence intensity of CD80 on DC was measured (*n* = 4). F–H) The number of immune cells in 5 × 10^5^ spinal cord cells analyzed by flow cytometry: macrophage number F), CD8^+^ T cell number G), and CD4^+^ T cell number H) (*n* = 5). At the end of the in vivo experiment, spinal cords were harvested and stained with various antibodies followed by analysis of immune cell profiles by flow cytometry. I) Plots showing IL‐17A^+^ CD4^+^ T cells in spinal cords. Cells from spinal cords were restimulated with 5 µg mL^−1^ of MOG_35–55_ and analyzed by flow cytometry. J) The percentage of IL‐17A^+^ CD4^+^ T cells in spinal cords (*n* = 3). K) The percentage of IFN‐*γ* ^+^ CD4^+^ T cells in spinal cords (*n* = 3). L) The frequency of CD25^+^ FOXP3^+^ T_reg_ cells among CD4^+^ T cells in spinal cords analyzed by flow cytometry (*n* = 5). M) Plots showing CD25^+^ FOXP3^+^ T_reg_ cells among CD4^+^ T cells in spinal cords. (Data are presented as the mean ± SD. *p < 0.05; **p < 0.01; ***p < 0.001).

Immunophenotyping of the CNS was subsequently conducted (**Figure** **8**A). Compared to subcutaneous AE treatment, AE/M administration via the same route significantly reduced CD86 and CD80 levels in DC by 1.6‐fold and 1.8‐fold, respectively (Figure 8B–E). Administration of AE/M notably diminished infiltration of macrophages into CNS when compared to all other groups. Specifically, in the AE/M‐treated group, macrophage infiltration was reduced by 6.8‐fold, relative to the AE treatment (Figure 8F). Furthermore, the AE/M treatment led to a decrease in the number of CD8^+^ and CD4^+^ T cells. This reduction was quantified as 8.0 times and 11.8 times lower, respectively, than that in the AE‐treated group (Figure 8G,H).

The treatment with AE/M effectively reduced the populations of IL‐17A^+^ CD4^+^ T cells and IFN‐*γ* ^+^ CD4^+^ T cells, with a reduction of 6.1‐fold and 2.7‐fold, respectively, compared to the AE‐treated group (Figures 8I–K and S14A, Supporting Information). Additionally, AE/M treatment significantly increased the population of CD25^+^ FOXP3^+^ T_reg_ cells, with a 5.0‐fold greater abundance than that in the AE‐treated group (Figure 8L,M). The T_reg_ cell population in the spinal cords of AE/M‐treated mice was comparable to that of normal controls, with no significant differences observed (Figure S14B, Supporting Information).

### 2.8 Histological Analysis of Leukocyte Infiltration and Demyelination in the CNS of EAE Mice

**Figure 9:**
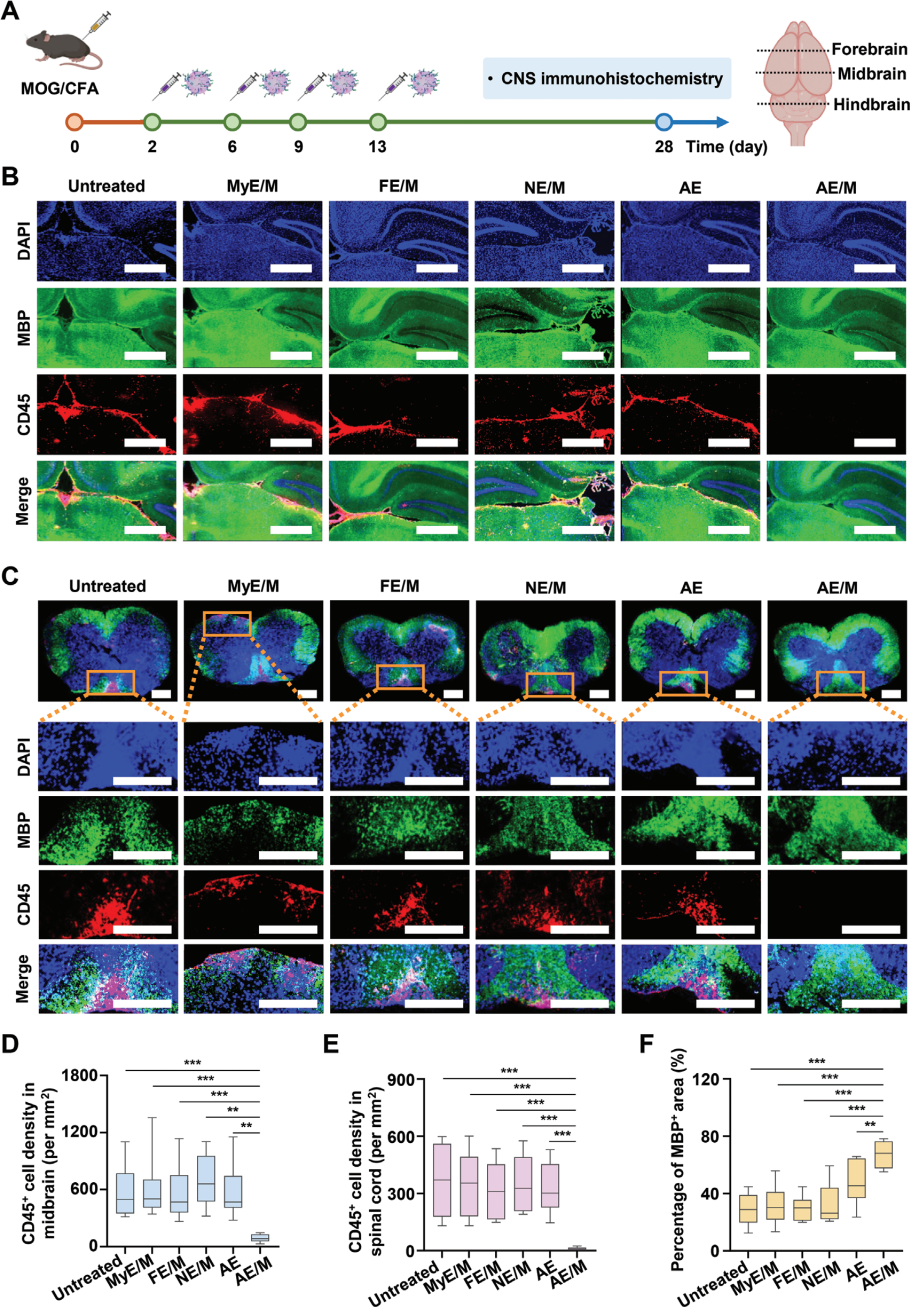
Infiltration of leukocytes to CNS. A) A schematic illustration depicts the schedule of the in vivo study. Subcutaneous administration of various exosome formulations began 2 days after EAE model induction. At the end of the study, brains and spinal cords were harvested to prepare tissue sections. B) Immunofluorescence images show leukocytes in the midbrain. Immunostaining was performed with DAPI (blue), anti‐MBP antibody (green), and anti‐CD45 antibody (red). Scale bar: 0.5 mm. C) Immunofluorescence images of leukocytes and demyelination in spinal cords, were analyzed using the THUNDER imaging system. Immunostaining was performed with DAPI (blue), anti‐MBP antibody (green), and anti‐CD45 antibody (red). Scale bar: 0.7 mm. D–E) The density of CD45^+^ cells in different CNS regions was analyzed using the Vectra tissue analyzer: midbrain D) and spinal cord E) (*n* = 10). F) The percentage of MBP^+^ area was calculated by dividing the MBP^+^ area by the total area of the selected tissue regions (*n* = 10). (Data are presented as the mean ± SD. **p < 0.01; ***p < 0.001).

Immunohistochemical analysis was used to assess leukocyte infiltration and demyelination in the CNS following four repeated subcutaneous administrations of MOG‐conjugated exosomes (**Figure** **9**A). AE/M treatment significantly reduced leukocyte infiltration in both the brain and spinal cord. Compared to the NE/M‐treated group, the AE/M‐treated group showed a substantial reduction in CD45^+^ cell density, with a 7.8‐fold reduction in the midbrain (Figure 9B), a 35.5‐fold reduction in the spinal cord (Figure 9C), a 4.8‐fold reduction in the forebrain, and a 15.2‐fold reduction in the hindbrain (Figure S15C–F, Supporting Information).

Similarly, AE/M treatment reduced CD45^+^ cell density compared to AE treatment, with a 6.5‐fold reduction in the midbrain (Figure 9D), a 32.5‐fold reduction in the spinal cord (Figure 9E), a 4.3‐fold reduction in the forebrain, and a 13‐fold reduction in the hindbrain (Figure S15C–F, Supporting Information). Demyelination of the spinal cord was observed in untreated samples and in those treated with MyE/M, FE/M, NE/M, and AE. AE/M treatment demonstrated a protective effect against spinal cord demyelination, as indicated by a 1.4‐fold increase in the myelin basic protein (MBP)‐positive area compared to AE treatment (Figure 9F).

## 3 Discussion

In this study, we developed immunomodulatory exosomes, AE/M which carried MOG antigen on their surface, promoting specific immune tolerance against self‐antigen. AE was found to induce tolDC by downregulating proinflammatory markers and upregulating anti‐inflammatory markers. In the EAE model, the administration of AE/M significantly protected mice from motor impairment and maintained neuro‐homeostasis. While this study demonstrated the natural targeting ability and immunomodulatory effects of AE using MOG as an antigen model, these exosomes can be utilized with different antigens, making them suitable for treating various autoimmune diseases.

The presence of CD9, CD63, and CD81, which are common surface markers of exosomes, was confirmed in all groups (Figure 2K,L). This observation validates the successful isolation of exosomes using the tangential flow filtration system.^[^ ^13^ ^]^ LFA‐1, an integrin found on leukocytes, was detected in NE/M and AE/M, but not in MyE/M and FE/M. This finding indicated variations in the natural properties of exosomes, reflecting the diversity of their donor cells. The exosomes were effectively decorated with cysteine‐modified MOG, as demonstrated by both confocal imaging and flow cytometry analysis. Crucially, this modification did not alter the size or surface charge of the exosomes, preserving their inherent biological properties (Figure 2A).

AE/M notably enhanced the expression of anti‐inflammatory markers while reducing proinflammatory markers compared to other exosomes tested (Figure 3H). This suggests that AE/M‐educated DC leans toward a tolerogenic phenotype, even when exposed to proinflammatory stimuli. It is important to note that exosomes carry the constituents of their originating cells, including proteins, DNA, and RNA.^[^ ^14^ ^]^ This implies that the biological properties of exosomes are inherited from their parent cells. The tolerogenic properties of AE/M may be derived from their parent cells, which are anti‐inflammatory macrophages. Anti‐inflammatory macrophages play a crucial role in resolving inflammation, primarily through the production of cytokines, such as IL‐10 and TGF‐*β*.^[^ ^15^, ^16^ ^]^

In the study, AE/M exosomes displayed a specific affinity for lymph nodes, accumulating predominantly in antigen‐presenting cells within these lymph nodes following subcutaneous administration (Figure 5). The results indicated that different types of exosomes can selectively bind to particular cells and target tissues similar to their source cells, a phenomenon influenced by the characteristics of the originating cells.^[^ ^17^, ^18^ ^]^ Supporting our findings, exosomes from immune cells have been previously reported to localize in secondary lymphoid organs.^[^ ^19^, ^20^ ^]^ Additionally, other studies have leveraged the natural cell‐targeting ability of exosomes for the focused delivery to tumor tissues using cancer cell‐derived exosomes.^[^ ^21^, ^22^, ^23^ ^]^

The targeting ability of AE/M toward antigen‐presenting cells may be largely due to their elevated expression of LFA‐1. The presence of LFA‐1 on AE and AE/M was evidenced in Figure 2K,L. Numerous studies have identified the presence of LFA‐1 in macrophage‐derived exosomes, playing a crucial role in their specific binding and uptake by antigen‐presenting cells.^[^ ^24^, ^25^, ^26^ ^]^ LFA‐1 in T cell exosomes has been reported as a vital factor in their uptake by DC.^[^ ^27^ ^]^ Based on these insights, it is hypothesized that LFA‐1 could facilitate the targeting of AE/M to DC by interacting with intercellular adhesion molecule‐1 expressed in these cells.

AE/M was found to effectively prevent the development of EAE in mice, with 100% of the mice remaining EAE‐free (Figure 6). AE/M also could avoid the issues of nonspecific immunosuppression or complete ineffectiveness, which were observed in groups treated with AE and NE/M alone (Figure 6B–D). The mechanism underlying this protection may involve DC presenting antigens without the costimulatory signals needed for full T cell activation, thereby inducing antigen‐specific immune tolerance. This process is a potent immune modulating strategy for autoimmune diseases.^[^ ^7^, ^28^ ^]^ Indeed, DC treated with AE/M displayed a tolerogenic phenotype and concurrently presented the self‐antigen MOG, leading to the establishment of MOG‐specific immune tolerance (Figure 7B–D).

The interaction between antigen‐presenting cells and naïve T cells in peripheral lymphoid organs, such as the lymph nodes and spleens, is crucial for determining the fate and immune function of T cells. When DC‐T cell interactions occur without costimulatory signals (e.g., interactions with tolDC) or in an environment where signals suppress costimulation, T cells can lose their effector functions and become anergic.^[^ ^29^ ^]^ Tolerogenic DC‐mediated peripheral tolerance is essential for preventing autoimmune diseases or hypersensitivity to antigens.^[^ ^7^ ^]^ In this study, we observed that AE/M treatment downregulated the expression of CD86 and CD80 in splenic DC, promoting the induction of anergic T cells (Figure 7E–J). The induction of anergic T cells has been reported to help prevent autoimmunity and give rise to T_reg_ cell precursors.^[^ ^30^ ^]^ The ability of AE/M to induce peripheral tolerance explains the promising prophylactic effects on EAE.

We observed that AE/M treatment established peripheral tolerance, as indicated by the induction of tolDC and anergic T cells in the spleens. This peripheral tolerance effectively eliminated autoreactive immune cells, resulting in reduced leukocyte infiltration into the CNS. In a healthy immune system, multiple tolerance mechanisms prevent autoimmunity, including central tolerance in the thymus and peripheral tolerance mediated by DC.^[^ ^7^ ^]^ Central tolerance occurs during T cell development in the thymus, while peripheral tolerance operates after central tolerance to prevent self‐reactive T and B cells that escape central tolerance from causing autoimmune diseases. Mechanisms such as anergy, deletion, and suppression by T_reg_ cells are critical for maintaining peripheral tolerance and preventing hypersensitivity to antigens encountered outside the thymus or bone marrow.^[^ ^29^ ^]^

In this study, we found that mice treated with AE/M maintained a balanced immune response in the CNS, leading to reduced tissue inflammation. The diminished inflammation in these AE/M‐treated mice appears to be due to a decrease in immune cells that could potentially damage the myelin sheaths surrounding neurons (Figure 8). CD11c^+^ DC in the CNS plays a pivotal role in restimulating antigen‐specific T cells and preparing them for tissue invasion, a process outlined by Hickey and Kimura,^[^ ^31^ ^]^ as well as Greter et al.^[^ ^32^ ^]^ The CD11c^+^ DC is capable of activating T cells that have infiltrated into CNS, leading to the initiation of epitope spreading, as described by McMahon et al.^[^ ^33^ ^]^ Notably, the AE/M treatment resulted in a significant decrease in the number of macrophages, CD8^+^ T cells, and CD4^+^ T cells. Additionally, AE/M intervention significantly reduced IL‐17A^+^ and IFN‐*γ* ^+^ CD4^+^ T cells that play a key role in disease pathology.^[^ ^34^ ^]^

In this study, we observed that AE/M treatment maintained T_reg_ cell populations in the CNS at levels similar to those in healthy controls (Figure S14B, Supporting Information). For T_reg_ cells to exert their suppressive function, controlling the local inflammatory environment in the target tissue is crucial. Previous reports have indicated that even if T_reg_ cells accumulate at the disease site, their suppressive function in the CNS may be compromised by the local inflammatory microenvironment.^[^ ^35^, ^36^ ^]^ When supportive factors for T_reg_ cell function, such as interleukin‐2, are absent or present at insufficient levels under inflammatory conditions, T_reg_ cells can become unstable and exhibit reduced FOXP3 levels,^[^ ^37^, ^38^ ^]^ as observed in all groups except the AE/M‐treated group. These data indicate that immune homeostasis may rely not only on the presence of T_reg_ cells but also on the functional balance between different immune cells. In this study, AE/M intervention preserved immune homeostasis in the CNS, as evidenced by reduced leukocyte infiltration and decreased production of pro‐inflammatory cytokines (Figure 8). By ensuring a balance between the regulatory and effector arms of the immune response, AE/M intervention effectively prevented the development of EAE.

The AE/M system shows promise for clinical relevance in the treatment of autoimmune diseases. First, AE/M may prove useful in modulating autoimmune responses during the early stages of the disease. The preclinical autoimmunity stage is characterized by detectable autoreactivity or autoimmunity in the absence of classic signs and symptoms of the disease. This stage eventually progresses to clinical autoimmune disease.^[^ ^39^ ^]^ Our observations suggest that AE/M‐mediated suppression of autoimmune responses at this early stage, prior to symptom onset, could effectively reduce both the clinical score and the rate of symptom progression.

Second, AE/M offers preventive potential by modulating underlying autoimmune responses in antigen‐specific immune tolerance, rather than merely alleviating symptoms. Current therapies for autoimmune conditions, including cytokines and small immunosuppressive molecules, are primarily aimed at symptom management. However, these treatments fail to address the underlying mechanisms driving autoimmune diseases.^[^ ^40^ ^]^

Third, AE/M may hold clinical significance due to its ability to avoid undesirable adverse effects. By inducing antigen‐specific immune tolerance, AE/M exerts a prophylactic effect without triggering broad, nonspecific immune suppression. In contrast, conventional therapies based on broad immunosuppressive mechanisms often result in adverse effects and increased susceptibility to infections.^[^ ^40^ ^]^

Despite its potential, the current AE/M approach has limitations. The progression of autoimmune diseases involves a diverse repertoire of epitopes on antigens, posing a challenge to the development of effective prophylactic treatments.^[^ ^41^ ^]^ In this study, a single antigen was targeted as proof of concept. Future research should focus on the discovery of diverse epitopes associated with autoimmune diseases and the development of delivery systems targeting multiple antigens. Combining the induction of immune tolerance to multiple representative antigens with AE should be explored to further enhance the clinical significance of AE‐based nanovaccines.

## 4 Conclusion

In this study, we developed an anti‐inflammatory macrophage exosome‐based nanoplatform for the prophylactic treatment of autoimmune diseases. Leveraging the natural properties of anti‐inflammatory macrophage exosomes, AE/M delivered the MOG self‐antigen to DCs, while simultaneously inducing tolDCs. This dual function enabled AE/M to establish immune tolerance, effectively preventing the development of EAE. In addition, AE/M demonstrated a promising effect in EAE mice by alleviating symptom severity. These findings highlight AE/M as a versatile treatment capable of addressing different stages of EAE progression, making it a promising candidate for autoimmune disease management. The preventive effects of AE/M could have clinical significance by reducing the onset and severity of symptoms through the modulation of antigen‐specific immune tolerance. Moreover, the ability of AE/M to induce antigen‐specific immune tolerance may mitigate the adverse effects associated with the nonspecific immune suppression caused by conventional immunosuppressive therapies. To advance AE‐based prophylactic vaccines for clinical applications, future research should focus on the delivery of diverse autoantigens to address the challenges posed by the complexity and heterogeneity of autoimmune diseases.

## 5 Experimental Section

### Exosome Isolation

To isolate exosomes, mouse Raw 264.7 cells, mouse myoblast C2C12 cells, and mouse fibroblast 3T3‐L1 cells (American Type Culture Collection, Blacksburg, VA) were used as donor cells. Donor cells were maintained in complete Dulbecco's modified Eagle's medium (DMEM, Welgene, Gyeongsangbuk‐do, Republic of Korea), which was supplemented with 100 units mL^−1^ of penicillin, 100 µg mL^−1^ of streptomycin (Capricorn Scientific GmbH, Hesse, Germany), and 10% heat‐inactivated bovine serum (Gibco, New York). To induce anti‐inflammatory macrophage phenotype, Raw 264.7 cells were treated with 20 ng mL^−1^ of IL‐4 (Cat# Z02996, GenScript, Piscataway, NJ) for 48 h, following previously reported protocols.^[^ ^19^, ^42^, ^43^ ^]^

Donor cells were allowed to grow for 2 days, after which the complete DMEM was replaced with serum‐free DMEM. Following an additional 2‐day incubation, the supernatant was collected through centrifugation at 2000 × g for 20 min. The filtered supernatant underwent sequential concentration using a tangential flow filtration system (Pall Corporation, Port Washington, NY) equipped with an ultrafiltration membrane filter capsule (MWCO = 300 kDa, Pall Corporation) to obtain exosomes. All exosome‐containing medium was washed with 12 mm phosphate‐buffered saline (1X PBS) for further experiments.

To isolate anti‐inflammatory macrophage‐derived exosomes, Raw 264.7 cells were first differentiated using complete DMEM, which included 20 ng mL^−1^ of IL‐4 (GenScript), as previously described. Following a 2‐day differentiation period, the differentiation medium was replaced with serum‐free DMEM and incubated for an additional 2 days. Subsequently, the medium containing anti‐inflammatory macrophage exosomes underwent processing through centrifugation and filtration, followed by concentration using a tangential flow filtration system.

### Conjugation of MOG Peptide to Exosomes

To conjugate the MOG_35–55_ peptide to the surface of exosomes, the N‐terminal cysteine‐modified MOG peptide (Cys‐MOG, CMEVGWYRSPFSRVVHLYRNGK, Peptron, Daejeon, Republic of Korea) (Figure S3A, Supporting Information) was utilized. The Cys‐MOG peptide contains a modified cysteine residue, which possesses a thiol group capable of reacting with maleimide. Myoblast exosomes, fibroblast exosomes, naïve macrophage exosomes, and anti‐inflammatory macrophage exosomes were allowed to react with maleimide‐(PEG)_2_‐NHS ester (TCI Chemicals, Tokyo, Japan) first (Figure S3B–E, Supporting Information). 0.3 mm of maleimide‐(PEG)_2_‐NHS ester was used to react with amine groups on the surface of exosomes at the concentration of 2.4 × 10^10^ mL^−1^. The reaction proceeded at room temperature for 4 h. To eliminate any unreacted maleimide‐(PEG)_2_‐NHS ester, a 100 kDa Amicon Ultra‐15 Centrifugal Filter (Merck Millipore, Burlington, MA) was utilized. Next, exosomes with a concentration of 1.2 × 10^11^ mL^−1^ were resuspended in PBS and allowed to react overnight with 0.08 mg mL^−1^ of Cys‐MOG (Figure S3B–E, Supporting Information). Unbound MOG was washed out, and the MOG‐conjugated exosomes were resuspended in 1X PBS.

MOG‐conjugated myoblast exosomes, MOG‐conjugated fibroblast exosomes, MOG‐conjugated naïve macrophage exosomes, and MOG‐conjugated anti‐inflammatory macrophage exosomes were obtained, depending on the donor cell types. The number of each MOG‐decorated exosome in PBS was quantified using nanoparticle tracking analysis (NanoSight LM10, Malvern Panalytical, Malvern, Worcestershire, UK).

### Physical Characterization of Exosomes

Various exosomes underwent characterization in terms of size distribution, zeta potential, and morphology. Size distribution and zeta potential of exosomes were determined using dynamic light scattering method and laser Doppler microelectrophoresis at an angle of 22° with an ELS8000 instrument (Photal, Osaka, Japan). Nanoparticle tracking analysis was employed to monitor the size distribution of various exosomes, conducted with NanoSight LM10 (Malvern Panalytical).

For the visualization of the 3D topography of exosomes, AFM was used. Exosomes were loaded onto poly‐L‐lysine coverslips (Corning, New York) and washed. The exosome‐bound coverslips were then allowed to dry overnight and analyzed using the Atomic Force Microscope AFM XE‐100 (Park Systems, Suwon, Republic of Korea).

TEM was used to visualize exosomes. Exosomes were adsorbed onto a grid and left to dry overnight. Negative staining was performed using 2% uranyl acetate for TEM observation.^[^ ^44^ ^]^ TEM images were captured using the Talos L120C microscope (Thermo Fisher, Waltham, MA). For SEM, exosomes were adsorbed onto aldehyde/sulfate latex beads (Invitrogen, Carlsbad, CA) overnight. Exosome‐bound beads were then placed onto carbon tape and allowed to dry overnight. Exosome morphology was examined using the Field‐Emission Scanning Electron Microscope (SIGMA) from Carl Zeiss (Oberkochen, Germany).

### Determination of Exosome Surface Markers

To verify the proper isolation of exosomes, exosome surface markers were examined and quantified using aldehyde/sulfate latex beads (Invitrogen) following a previously reported protocol.^[^ ^45^ ^]^ In brief, exosome samples were mixed with beads and left overnight. Subsequently, the exosome‐bound beads were collected through centrifugation and resuspended in 500 mm of glycine (Sigma‐Aldrich, St. Louis, MO) to block any remaining binding sites on the aldehyde/sulfate latex beads for 1 h.

Next, the beads were stained with fluorescein isothiocyanate (FITC)‐conjugated antimouse CD9 antibody (1:100, Cat# 124 807, BioLegend, San Diego, CA), phycoerythrin (PE)‐conjugated antimouse CD81 antibody (1:100, Cat# 104 905, BioLegend), PerCP/Cyanine5.5‐conjugated antimouse CD63 antibody (1:100, Cat# 143 911, BioLegend), and allophycocyanin (APC)‐conjugated antimouse LFA‐1 antibody (1:100, Cat# 141 009, BioLegend) for 1 h at room temperature.

To detect surface‐conjugated MOG peptide, beads were initially labeled with the primary mouse anti‐MOG antibody (1:50, Cat# sc‐73330, Santa Cruz Biotechnology, Dallas, TX), followed by a second staining step using Alexa Fluor 647‐conjugated antimouse IgG antibody (1:100, Cat# 405 322, BioLegend). The expression of surface markers was analyzed using BD FACSLyric Flow Cytometry System (BD Biosciences, San Jose, CA). Relative mean fluorescence intensity (Relative MFI) was determined by comparing the mean fluorescence intensity of exosome‐bound aldehyde/sulfate latex beads to that of blank aldehyde/sulfate latex beads. For visualization of surface markers, antibody‐stained beads were plated onto poly‐L‐lysine coverslips (Corning) and allowed to attach for 1 h. Fluorescence was observed using Confocal Scope TCS8 (Leica, Wetzlar, Germany). Quantitative analysis of fluorescence intensity was conducted using ImageJ software (National Institutes of Health, Bethesda, MD).

### Quantification of MOG Peptide Conjugated to Exosomes

The amount of MOG peptide conjugated to the surfaces of exosomes was determined using HPLC (Hewlett Packard model 1100 system, Hewlett Packard, Palo Alto, CA) as described previously.^[^ ^46^ ^]^ After conjugation, the reacted samples were loaded into a 50 kDa Amicon Ultra‐0.5 filter (Merck Millipore) and centrifuged at 14 000 × g for 5 min. A mixture of acetonitrile, water, and trifluoroacetic acid (30:70:0.1, v:v:v) was used as the mobile phase. The flow rate was maintained at 1.0 mL min^−1^, and the MOG peptide was monitored at an absorbance of 220 nm. Solutions containing unconjugated MOG were loaded onto a reverse‐phase C18 HPLC column (Phenomenex, Torrance, CA). The conjugation efficiency of MOG was calculated as follows

(1) $$\begin{array}{ccc} & & \mathrm{Conjugation}\;\mathrm{efficiency}\;\left(\%\right)\\ & & \;=\frac{\mathrm{Total}\;\mathrm{amount}\;\mathrm{of}\;\mathrm{MOG}\;-\;\mathrm{Unconjugated}\;\mathrm{amount}\;\mathrm{of}\;\mathrm{MOG}}{\mathrm{Total}\;\mathrm{amount}\;\mathrm{of}\;\mathrm{MOG}}\times 100 \end{array}$$

### In Vitro Induction of TolDC

The induction of tolDC by exosomes was assessed using splenocytes and quantified with BD FACSLyric Flow Cytometry System (BD Biosciences). Spleens were collected from C57BL/6 mice and processed into single cells, excluding red blood cells using ACK lysis buffer (Gibco). The isolated splenocytes were then incubated with various exosome formulations at a dose of 6 × 10^9^ exosomes mL^−1^ for 72 h. To evaluate the expression levels of CD86, CD80, MHCII, and PD‐L1, splenocytes were exposed to 0.5 µg mL^−1^ of lipopolysaccharide (LPS, Sigma‐Aldrich) for 24 h. For assessing the expression levels of TNF‐*α*, IL‐6, IL‐10, and TGF‐*β*, splenocytes were treated with 1 µg mL^−1^ of LPS (Sigma‐Aldrich) and 5 µg mL^−1^ of brefeldin A (Invitrogen) for 6 h.

To analyze the expression of tolDC‐related markers, splenocytes were labeled with the following antibodies: FITC‐conjugated antimouse CD11c antibody (1:100, Cat# 117 306, BioLegend), PerCP/Cyanine5.5‐conjugated antimouse I‐A/I‐E antibody (1:100, Cat# 107 626, BioLegend), PE‐conjugated antimouse CD86 antibody (1:100, Cat# 105 008, BioLegend), APC‐conjugated antimouse CD80 antibody (1:100, Cat# 104 714, BioLegend), and APC‐conjugated antimouse PD‐L1 antibody (1:100, Cat# 124 311, BioLegend).

Intracellular staining was performed for cytokine detection using a True Nuclear Transcription Factor Buffer Set (BioLegend). Briefly, splenocytes were labeled with FITC‐conjugated antimouse CD11c antibody (1:100, Cat# 117 306, BioLegend) and PerCP/Cyanine5.5‐conjugated antimouse I‐A/I‐E antibody (1:100, Cat# 107 626, BioLegend) for 1 h, followed by fixation with fixation buffer. Intracellular staining was carried out using PE‐conjugated antimouse TNF‐*α* antibody (1:100, Cat# 506 306, BioLegend), APC‐conjugated antimouse IL‐6 antibody (1:100, Cat# 504 507, BioLegend), APC‐conjugated antimouse IL‐10 antibody (1:100, Cat# 505 009, BioLegend), and APC‐conjugated antimouse TGF‐*β* antibody (1:100, Cat# 141 405, BioLegend). Quantification of tolDC‐related marker expression was performed using BD FACSLyric Flow Cytometry System (BD Biosciences). To visualize marker expression, BMDC were incubated with AE/M at a concentration of 6 × 10⁹ mL^−1^ for 24 h, with 0.5 µg mL^−1^ of LPS added to the cells. BMDC was stained with Alexa Fluor 594‐conjugated antimouse CD11c antibody (1:100, Cat# 117 346, BioLegend), FITC‐conjugated antimouse I‐A/I‐E antibody (1:100, Cat# 107 605, BioLegend), PE‐conjugated antimouse CD86 antibody (1:100, Cat# 105 008, BioLegend), APC‐conjugated antimouse CD80 antibody (1:100, Cat# 104 714, BioLegend), and APC‐conjugated antimouse PD‐L1 antibody (1:100, Cat# 124 311, BioLegend). The visualization was achieved using the Confocal Scope TCS8 (Leica).

### Assessment of Exosome‐Induced Changes on BMDC and BMDM

The effect of exosomes on BMDC and BMDM was investigated as previously described, with slight modifications.^[^ ^47^, ^48^ ^]^ BMDM and BMDC were cultured as previously described.^[^ ^49^ ^]^ BMDC and BMDM were incubated with various exosomes at a concentration of 6 × 10⁹ mL^−1^ for 24 h, with 0.5 µg mL^−1^ of LPS added to the cells. Both cells and culture medium were collected for further analysis.

To analyze CD86 expression, BMDC was stained with FITC‐conjugated antimouse CD11c antibody (1:100; Cat# 117 306, BioLegend), PerCP/Cyanine5.5‐conjugated antimouse I‐A/I‐E antibody (1:100; Cat# 107 626, BioLegend), and PE‐conjugated antimouse CD86 antibody (1:100; Cat# 105 008, BioLegend). BMDM was labeled with PE‐conjugated antimouse CD86 antibody (1:100; Cat# 105 008, BioLegend) and APC/Cyanine7‐conjugated anti‐mouse F4/80 antibody (1:100; Cat# 123 118, BioLegend).

To measure intracellular ROS in BMDM, cells were stained with CM‐H2DCFDA (Invitrogen) according to the manufacturer's instructions. Fluorescence was acquired using the BD FACSLyric Flow Cytometry System (BD Biosciences). The level of TNF‐*α* in the culture medium was determined using a TNF‐*α* enzyme‐linked immunosorbent assay (ELISA) kit (R&D Systems, Minneapolis, MN) following the manufacturer's instructions.

### Cellular Uptake of Exosomes

Cellular uptake of fluorescent dye‐labeled exosomes was assessed using mouse BMDM and BMDC. To label MyE/M, FE/M, NE/M, AE, and AE/M, they were stained with 10 µm of DiD (Invitrogen) for 1 h, followed by three washes with 100 kDa Amicon Ultra‐15 Centrifugal Filter (Merck Millipore). Subsequently, the DiD‐labeled exosomes were added to BMDM or BMDC and allowed to incubate for 1 h at 37 °C. BMDM and BMDC were stained with PE‐conjugated antimouse F4/80 antibody (1:100, Cat# 123 110, BioLegend) and PE‐conjugated antimouse CD11c antibody (1:100, Cat# 117 308, BioLegend), respectively. The cellular uptake of each exosome was analyzed using BD FACSLyric Flow Cytometry System (BD Biosciences).

To visualize the cellular uptake, DiD‐labeled exosomes were treated to BMDM or BMDC. Subsequently, BMDM and BMDC were stained with PE‐conjugated antimouse F4/80 antibody (1:100, Cat# 123 110, BioLegend) and PE‐conjugated antimouse CD11c antibody (1:100, Cat# 117 308, BioLegend) for 1 h, respectively. After surface staining, cells were fixed with 4% paraformaldehyde for another 20 min followed by nuclei labeling with 1 µg mL^−1^ of DAPI (Sigma‐Aldrich). Cellular fluorescence was observed using Confocal Scope TCS8 (Leica).

Cellular uptake of AE/M at different time points was visualized by Confocal Scope TCS8 (Leica). AE/M was labeled with 10 µm of DiD (Invitrogen) and treated to BMDC for 1, 2, and 4 h. After incubation, BMDC was labeled with FITC‐conjugated antimouse CD11c antibody (1:100, Cat# 117 306, BioLegend) for 1 h. The fixation was performed with 4% paraformaldehyde for another 20 min. Nucleus was labeled with DAPI (Sigma‐Aldrich) at a concentration of 1 µg mL^−1^.

To further study the cellular uptake of exosomes in antigen‐presenting cells from EAE mice, spleens were harvested and processed into splenocytes. DiD‐labeled exosomes were added to the splenocytes and incubated for 1 h at 37 °C. Next, the splenocytes were stained with FITC‐conjugated antimouse CD11c antibody (1:100; Cat# 117 306, BioLegend), PerCP/Cyanine5.5‐conjugated antimouse I‐A/I‐E antibody (1:100; Cat# 107 626, BioLegend), and Alexa Fluor 700‐conjugated antimouse F4/80 antibody (1:100; Cat# 123 130, BioLegend). Fluorescence signals in the cells were assessed using the BD FACSLyric Flow Cytometry System (BD Biosciences).

### Animals

Twelve‐week‐old C57BL/6 mice were obtained from Raon Bio (Raon Bio Korea, Yongin, Republic of Korea) and were kept in a standard pathogen‐free environment at the Animal Center for Pharmaceutical Research, Seoul National University. All experiments were conducted following the Guidelines for the Care and Use of Laboratory Animals of the Institute of Laboratory Animal Resources, Seoul National University (Approval number: SNU‐210106‐4).

### Biodistribution Study

To investigate the in vivo distribution patterns of exosomes derived from different sources, a biodistribution study was conducted using DiD‐labeled exosomes. These DiD‐labeled exosomes were subcutaneously injected at the center of the lower back of C57BL/6 mice, at a dosage of 2.4 × 10^10^ DiD‐labeled exosomes (equivalent to 1.2 × 10^12^ exosomes kg^−1^). Mice were euthanized at various time points after administration. The fluorescence signals in various organs were detected using an IVIS Spectrum CT In Vivo Imaging System (PerkinElmer, Waltham, MA).

### Visualization of Exosomes in Lymph Nodes

To visualize the distribution of exosomes in lymph nodes, DiD‐labeled exosomes were subcutaneously administered to C57BL/6 mice at a dosage of 2.4 × 10^10^ exosomes (equivalent to 1.2 × 10^12^ exosomes kg^−1^). After 24 h, lymph nodes were collected, fixed with 10% formalin for 24 h, and then immersed in 30% sucrose for an additional 48 h. Cryosections of the lymph nodes were prepared and incubated with FITC‐conjugated antimouse CD11c antibody (1:100, Cat# 117 306, BioLegend) and PE‐conjugated antimouse F4/80 antibody (1:100, Cat# 123 110, BioLegend) overnight at 4 °C. Following immunostaining, the lymph node sections were stained with 2 µg mL^−1^ of DAPI for 40 min and examined using a THUNDER imaging system (Leica).

### Distribution of Exosomes in Immune Cells Isolated from Lymph Nodes

To explore the internalization of exosomes by immune cells in lymph nodes, DiD‐labeled exosomes were subcutaneously administered to C57BL/6 mice at a dosage of 2.4 × 10^10^ exosomes (equivalent to 1.2 × 10^12^ exosomes kg^−1^). After 24 h, lymph nodes were harvested and processed through cell strainers (70 µm, SPL Life Sciences, Pocheon, Republic of Korea) to create a single‐cell suspension. These cells were then labeled with FITC‐conjugated antimouse CD11c antibody (1:100, Cat# 117 306, BioLegend), PE‐conjugated antimouse CD19 antibody (1:100, Cat# 115 507, BioLegend), Alexa Fluor 700‐conjugated antimouse F4/80 antibody (1:100, Cat# 123 130, BioLegend), and PerCP/Cyanine5.5‐conjugated antimouse I‐A/I‐E antibody (1:100, Cat# 107 626, BioLegend). The fluorescence signals in the cells were assessed using BD FACSLyric Flow Cytometry System (BD Biosciences).

### In Vivo Efficacy Study

The in vivo efficacy of various exosome formulations was assessed using an EAE mouse model, which simulates the pathology of human multiple sclerosis.^[^ ^50^ ^]^ In brief, an equivalent volume of 2 mg mL^−1^ MOG_35–55_ peptides (Prospec, Rehovot, Israel) and Complete Freund's Adjuvant (CFA), containing 4 mg mL^−1^ of heat‐killed Mycobacterium tuberculosis H37RA (Chondrex, Woodinville, WA), was mixed using a homogenizer (Qiagen, Hilden, Germany). The emulsified MOG peptides were administered subcutaneously at a dose of 200 µg per mouse. In addition, 400 ng of pertussis toxin (List Biological Labs, Campbell, CA) was intraperitoneally administered 2 and 24 h after emulsion injection.

C57BL/6 mice received various exosome formulations subcutaneously at a dose of 2.4 × 10^10^ (equivalent to 1.2 × 10^12^ exosomes kg^−1^) every 3 or 4 days, starting from day 2, for a total of 4 administrations. Clinical score and body weight were recorded.

In vivo efficacy of AE/M was also assessed through intravenous administration. C57BL/6 mice received various exosome formulations at a dose of 2.4 × 10^10^ exosomes (equivalent to 1.2 × 10^12^ exosomes kg^−1^) every 3 or 4 days, starting on day 2 after MOG peptide immunization, for a total of 4 administrations. Clinical score and body weight were monitored throughout the in vivo study.

To assess the in vivo effect, exosomes were subcutaneously administered after the onset of EAE (day 9) at a dose of 2.4 × 10¹⁰ exosomes (equivalent to 1.2 × 10¹^2^ exosomes kg^−1^). The exosomes were administered for a total of 4 administrations. Clinical score and body weight were monitored throughout the in vivo study.

Clinical symptoms were assessed using a 0–5 scale as follows: 0 for no obvious change in motor function compared to nonimmunized mice, 1 for limp tail, 2 for limp tail and weakness of hind legs, 3 for limp tail and complete paralysis of hind legs, 4 for limp tail, complete hind leg and partial front leg paralysis, and 5 for death or euthanasia due to severe paralysis.^[^ ^50^ ^]^

To assess antigen‐specific immune responses, an ELISpot assay and ELISA were conducted. In brief, isolated splenocytes (2 × 10^6^) were reactivated with 5 µg mL^−1^ of MOG_35‐55_ peptides for 24 h, and the spots of IFN‐*γ*‐secreting cells were quantified using an ELISpot kit (BD Biosciences). The antigen‐specific immune response was also determined through an IL‐17 secretion determination. Splenocytes (2 × 10^6^) were isolated from each mouse at the end of the study and stimulated with 10 µg mL^−1^ of MOG_35–55_ peptides for 72 h. The amount of secreted IL‐17 was quantified using an IL‐17 ELISA kit (R&D Systems) following the manufacturer's instructions.

### Evaluation of In Vivo Induction of Immune Tolerance

To evaluate peripheral tolerance in the spleens, immune cell profiling was performed using flow cytometry on a BD FACSLyric Flow Cytometry System (BD Biosciences). For the analysis of anergic T cells, splenocytes were stained with the following antibodies: FITC‐tagged antimouse CD44 antibody (1:100; Cat#156 008, BioLegend), PE‐tagged antimouse CD4 antibody (1:100; Cat#100 407, BioLegend), PerCP/Cyanine5.5‐tagged antimouse FR4 antibody (1:100; Cat#125 017, BioLegend), APC‐tagged antimouse FOXP3 antibody (1:100; Cat#17‐5773‐82, Invitrogen), and APC/Cyanine7‐tagged antimouse CD73 antibody (1:100; Cat#127 231, BioLegend). Anergic T cells were identified as previously described,^[^ ^30^, ^51^ ^]^ and the gating strategy is shown in Figure S16A (Supporting Information).

To investigate DC phenotypes in the spleens, splenocytes were labeled with FITC‐conjugated antimouse CD11c antibody (1:100, Cat# 117 306, BioLegend), PerCP/Cyanine5.5‐conjugated antimouse I‐A/I‐E antibody (1:100, Cat# 107 626, BioLegend), PE‐conjugated antimouse CD86 antibody (1:100, Cat# 105 008, BioLegend), and APC‐conjugated antimouse CD80 antibody (1:100, Cat# 104 714, BioLegend). For DC identification, the gating strategy is shown in Figure S16B (Supporting Information).

### Analysis of Leukocyte Infiltration and DC Phenotyping in Spinal Cords

Immune cell populations in the spinal cords of EAE and normal mice were assessed using flow cytometry analysis. After 28 days following the initial exposure to the MOG_35–55_ peptide, the spinal cord tissues were homogenized, and individual cells were obtained by incubating them in RPMI1640 medium supplemented with 1 mg mL^−1^ of collagenase (Sigma‐Aldrich). This mixture was incubated at 37 °C for 1 h. After the digestion process, the cells were washed using PBS containing 2% fetal bovine serum, and red blood cells were removed by treating the cells with ACK lysis buffer for 3 min.

The spinal cord cell suspension was stained with PerCP/Cyanine5.5‐tagged antimouse CD45 antibody (1:100, Cat#103 132, BioLegend), PE‐tagged antimouse CD11b antibody (1:100, Cat#123 110, BioLegend), APC‐tagged antimouse F4/80 antibody (1:100, Cat#123 115, BioLegend), FITC‐tagged antimouse CD3e antibody (1:100, Cat#100 204, BioLegend), APC‐tagged antimouse CD8a antibody (1:100, Cat#100 306, BioLegend), PE‐tagged antimouse CD4 antibody (1:100, Cat#100 407, BioLegend), FITC‐tagged antimouse CD25 antibody (1:100, Cat#102 006, BioLegend), and APC‐tagged antimouse FOXP3 antibody (1:100, Cat#17‐5773‐82, Invitrogen). The number of macrophages (CD45^+^ CD11b^+^ F4/80^+^), CD8^+^ T cells (CD45^+^ CD3^+^ CD8^+^), CD4^+^ T cells (CD45^+^ CD3^+^ CD4^+^), and the frequency of T_reg_ cells (CD45^+^ CD4^+^ CD25^+^ FOXP3^+^) were analyzed using a BD FACS Calibur flow cytometer (BD Biosciences). To analyze IL‐17A and IFN‐*γ*‐expressing T cells, the spinal cord cell suspension was treated with 5 µg mL^−1^ of MOG_35‐55_ peptide and 5 µg mL^−1^ of brefeldin A for 24 h. Intracellular staining was conducted with APC‐tagged antimouse IL‐17A antibody (1:100, Cat#505 810, BioLegend) or APC‐tagged antimouse IFN‐*γ* antibody (1:100, Cat#506 916, BioLegend). CD45^+^ CD3^+^ CD4^+^ T cells were selected as CD4^+^ T cells, and the expression of IL‐17A and IFN‐*γ* was analyzed using a BD FACS Calibur flow cytometer (BD Biosciences). The leukocytes in the CNS were identified as described previously,^[^ ^50^, ^52^ ^]^ with the gating strategy for leukocytes presented in Figure S17 (Supporting Information).

To analyze DC phenotypes in the spinal cords, leukocytes were isolated using a Percoll density gradient method with slight modifications as previously described.^[^ ^53^, ^54^ ^]^ Briefly, spinal cord cells were suspended in 70% Percoll. Then, 37% Percoll and 30% Percoll were layered onto the 70% Percoll cell suspension. The suspension was centrifuged at 800 × *g* for 25 min, and leukocytes were collected from the interface between the 37% and 70% Percoll layers. These leukocytes were further centrifuged at 400 × *g* for 7 min to obtain a purified leukocyte population.

To investigate DC phenotypes, the isolated leukocytes were labeled with FITC‐tagged antimouse CD44 antibody (1:100, Cat#156 008, BioLegend), PE‐tagged antimouse CD11c antibody (1:100, Cat#117 308, BioLegend), PerCP/Cyanine5.5‐tagged antimouse CD45 antibody (1:100, Cat#103 132, BioLegend), Brilliant Violet 421‐tagged antimouse I‐A/I‐E antibody (1:100, Cat#107 631, BioLegend), APC‐tagged antimouse CD80 antibody (1:100, Cat#104 714, BioLegend), and PE/Cyanine7‐tagged antimouse CD86 antibody (1:100, Cat#159 207, BioLegend). Given that microglia have lower CD45 expression compared to leukocytes, microglia were excluded based on CD45 levels, while dendritic cells were identified as described previously.^[^ ^55^, ^56^, ^57^ ^]^ The gating strategy is provided in Figure S17B (Supporting Information).

### Immunohistochemistry Study

For immunofluorescence staining, the isolated spinal cords and brains underwent fixation in 10% formalin for 24 h. To prevent tissue damage during freezing, the samples were immersed in a 30% sucrose/PBS solution w/v until the organs settled. The fixed tissues were embedded in Tissue‐Tek O.C.T. medium (Sakura Finetek, Torrance, CA). Sections measuring 8 µm in thickness were subjected to a blocking step using a 1% bovine serum albumin solution. Subsequently, these sections were exposed to staining agents: Alexa Fluor 647‐tagged antimouse CD45 antibody (1:100, Cat#103 124, BioLegend) and Alexa Fluor 488‐tagged antimouse Myelin Basic Protein antibody (1:100, Cat#850 908, BioLegend). The sections of spinal cord and brain tissues were visualized using a THUNDER imaging system (Leica) and analyzed with the Vectra Automated Multimodal Tissue Analysis System (PerkinElmer).

To quantify the MBP^+^ area in spinal cords, regions of interest were randomly selected within the white matter and analyzed using ImageJ (National Institutes of Health). The total area of the selected tissue was measured by applying a threshold to the DAPI staining. The MBP^+^ area was then determined by thresholding the MBP staining and expressed as a proportion of the total selected tissue area, as previously described with slight modifications.^[^ ^58^ ^]^

### Statistical Analysis

Data are presented as the mean ± standard deviation or as box‐and‐whisker plots of medians with minimum–maximum. Statistical analysis between two groups was performed using the Student's *t*‐test. For analysis of more than two groups, a one‐way analysis of variance (ANOVA) with Tukey's test for post‐hoc analysis was employed to assess the statistical significance of the experimental data. Statistical differences were calculated using GraphPad Prism software (v8.0, GraphPad Software, San Diego, CA). A *P*‐value less than 0.05 was considered statistically significant.

## Conflict of Interest

The authors declare no conflict of interest.

## Author Conbributions

Q.L. and J.P. contributed equally to this work.

## Supporting information



Supporting Information

Supplemental Video 1

Supplemental Video 2A

Supplemental Video 2B

## Data Availability

The data that support the findings of this study are available from the corresponding author upon reasonable request.

## References

[1] N. Theofilopoulos , D. H. Kono , R. Baccala , Nat. Immunol. 2017, 18, 716. 28632714 10.1038/ni.3731 PMC5791156

[2] K. L. Heckman , A. Y. Estevez , W. DeCoteau , S. Vangellow , S. Ribeiro , J. Chiarenzelli , B. Hays‐Erlichman , J. S. Erlichman , Front. Pharmacol. 2020, 10, 1599. 32047435 10.3389/fphar.2019.01599 PMC6997543

[3] G. Di Mauro , R. Amoriello , N. Lozano , A. Carnasciali , D. Guasti , M. Becucci , G. Cellot , K. Kostarelos , C. Ballerini , L. Ballerini , ACS Nano 2023, 17, 1965. 36692902 10.1021/acsnano.2c06609 PMC9933621

[4] M. Aldayel , H. L. O'Mary , S. A. Valdes , X. Li , S. G. Thakkar , B. E. Mustafa , Z. Cui , J. Controlled Release 2018, 283, 280. 10.1016/j.jconrel.2018.05.035 PMC6092926 29859232

[5] W. M. Stauffer , J. D. Alpern , P. F. Walker , JAMA, J. Am. Med. Assoc. 2020, 324, 623. 10.1001/jama.2020.13170 32761166

[6] Y. Zhou , Z. Peng , E. S. Seven , R. M. Leblanc , J. Controlled Release 2018, 270, 290. 10.1016/j.jconrel.2017.12.015 29269142

[7] A. D. Cifuentes‐Rius , D. Yuen , A. P. R. Johnston , N. H. Voelcker , Nat. Nanotechnol. 2021, 16, 37. 33349685 10.1038/s41565-020-00810-2

[8] K. Herrmann , M. J. A. Wood , G. Fuhrmann , Nat. Nanotechnol. 2021, 16, 748. 34211166 10.1038/s41565-021-00931-2

[9] R. Cecchin , Z. Troyer , K. Witwer , K. V. Morris , Mol. Ther. 2023, 31, 1225. 36698310 10.1016/j.ymthe.2023.01.021 PMC10188631

[10] Y. Wang , M. Zhao , S. Liu , J. Guo , Y. Lu , J. Cheng , J. Liu , Cell Death Dis. 2020, 11, 924. 33116121 10.1038/s41419-020-03127-z PMC7595091

[11] D. G. Russell , L. Huang , B. C. VanderVen , Nat. Rev. Immunol. 2019, 19, 291. 30679807 10.1038/s41577-019-0124-9 PMC7032560

[12] Y. Tang , Z. Wu , R. Guo , J. Huang , X. Rong , B. Zhu , L. Wang , L. Ma , C. Cheng , L. Qiu , J. Mater. Chem. B 2022, 10, 7862. 36070446 10.1039/d2tb01219g

[13] A. F. H. Lötvall , F. Hochberg , E. I. Buzás , D. Di Vizio , C. Gardiner , Y. S. Gho , I. V. Kurochkin , S. Mathivanan , P. Quesenberry , S. Sahoo , H. Tahara , M. H. Wauben , K. W. Witwer , C. Théry , J. Extracell. Vesicles 2014, 3, 26913. 25536934 10.3402/jev.v3.26913 PMC4275645

[14] R. Kalluri , V. S. LeBleu , Science 2020, 367, eaau6977. 32029601 10.1126/science.aau6977 PMC7717626

[15] S. Pérez , S. Rius‐Pérez , Antioxidants 2022, 11, 1394. 35883885 10.3390/antiox11071394 PMC9311967

[16] S. M. Shapouri‐Moghaddam , H. Vazini , M. Taghadosi , S. A. Esmaeili , F. Mardani , B. Seifi , A. Mohammadi , J. T. Afshari , A. Sahebkar , J. Cell. Physiol. 2018, 233, 6425. 10.1002/jcp.26429 PMC13482560 29319160

[17] T. Wan , J. Zhong , Q. Pan , T. Zhou , Y. Ping , X. Liu , Sci. Adv. 2022, 8, eabp9435. 36103526 10.1126/sciadv.abp9435 PMC9473578

[18] X. Zheng , K. Sun , Y. Liu , X. Yin , H. Zhu , F. Yu , W. Zhao , J. Controlled Release 2023, 353, 675. 10.1016/j.jconrel.2022.12.026 36521687

[19] Y. W. Cheng , L. Huang , Mol. Ther. 2017, 25, 1665. 28284981 10.1016/j.ymthe.2017.02.007 PMC5498801

[20] O. P. Wiklander , J. Z. Nordin , A. O'Loughlin , Y. Gustafsson , G. Corso , I. Mäger , P. Vader , Y. Lee , H. Sork , Y. Seow , N. Heldring , L. Alvarez‐Erviti , C. I. Smith , K. L.e Blanc , P. Macchiarini , P. Jungebluth , M. J. Wood , S. E. Andaloussi , J. Extracell. Vesicles 2015, 4, 26316. 25899407 10.3402/jev.v4.26316 PMC4405624

[21] M. Kim , Y. Yang , S. J. Oh , Y. Hong , M. Seo , M. Jang , J. Controlled Release 2017, 266, 8. 10.1016/j.jconrel.2017.09.013 28916446

[22] E. Emam , A. S. Abu Lila , N. E. Elsadek , H. Ando , T. Shimizu , K. Okuhira , Y. Ishima , M. A. Mahdy , F. S. Ghazy , T. Ishida , Eur. J. Pharm. Biopharm. 2019, 145, 27. 31629787 10.1016/j.ejpb.2019.10.005

[23] Y. J. Li , J. Y. Wu , J. M. Wang , X. B. Hu , J. X. Cai , D. X. Xiang , Acta Biomater. 2020, 101, 519. 31629893 10.1016/j.actbio.2019.10.022

[24] D. Yuan , Y. Zhao , W. A. Banks , K. M. Bullock , M. Haney , E. Batrakova , A. V. Kabanov , Biomaterials 2017, 142, 1. 28715655 10.1016/j.biomaterials.2017.07.011 PMC5603188

[25] H. Li , Y. Feng , X. Zheng , M. Jia , Z. Mei , Y. Wang , Z. Zhang , M. Zhou , C. Li , J. Controlled Release 2022, 341, 16. 10.1016/j.jconrel.2021.11.019 34793917

[26] N. Zhang , Y. Song , Z. Huang , J. Chen , H. Tan , H. Yang , M. Fan , Q. Li , Q. Wang , J. Gao , Z. Pang , J. Qian , J. Ge , Biomaterials 2020, 255, 120168. 32562944 10.1016/j.biomaterials.2020.120168

[27] H. Z. Xie , W. Li , Y. Deng , M. A. Munegowda , R. Chibbar , M. Qureshi , J. Xiang , J. Immunol. 2010, 185, 5268. 20881190 10.4049/jimmunol.1000386

[28] S. D. Miller , D. M. Turley , J. R. Podojil , Nat. Rev. Immunol. 2007, 7, 665. 17690713 10.1038/nri2153

[29] J. E. Kenison , N. A. Stevens , F. J. Quintana , Nat. Rev. Immunol. 2024, 24, 338. 38086932 10.1038/s41577-023-00970-x PMC11145724

[30] S. E. S. Kalekar , S. L. Nandiwada , W. Y. Lam , L. O. Barsness , N. Zhang , G. L. Stritesky , D. Malhotra , K. E. Pauken , J. L. Linehan , M. G. O'Sullivan , B. T. Fife , K. A. Hogquist , M. K. Jenkins , D. L. Mueller , Nat. Immunol. 2016, 17, 304. 26829766 10.1038/ni.3331 PMC4755884

[31] W. F. Hickey , H. Kimura , Science 1988, 239, 290. 3276004 10.1126/science.3276004

[32] F. L. H. Greter , M. P. Lemos , B. M. Odermatt , N. Goebels , T. Laufer , R. J. Noelle , B. Becher , Nat. Med. 2005, 11, 328. 15735653 10.1038/nm1197

[33] E. J. McMahon , S. L. Bailey , C. V. Castenada , H. Waldner , S. D. Miller , Nat. Med. 2005, 11, 335. 15735651 10.1038/nm1202

[34] M. McGinley , S. C. Edwards , M. Raverdeau , K. H. G. Mills , J. Autoimmun. 2018, 87, 97. 10.1016/j.jaut.2018.01.001 29395738

[35] J. Schlöder , F. Shahneh , F. J. Schneider , B. Wieschendorf , Front. Immunol. 2022, 13, 973813. 36032121 10.3389/fimmu.2022.973813 PMC9400058

[36] T. Korn , J. Reddy , W. Gao , E. Bettelli , A. Awasthi , T. R. Petersen , B. T. Bäckström , R. A. Sobel , K. W. Wucherpfennig , T. B. Strom , M. Oukka , V. K. Kuchroo , Nat. Med. 2007, 13, 423. 17384649 10.1038/nm1564 PMC3427780

[37] T. S. Sumida , N. T. Cheru , D. A. Hafler , Nat. Rev. Immunol. 2024, 24, 503. 38374298 10.1038/s41577-024-00994-x PMC11216899

[38] D. A. H. Dominguez‐Villar , Nat. Immunol. 2018, 19, 665. 29925983 10.1038/s41590-018-0120-4 PMC7882196

[39] D. S. Pisetsky , Nat. Rev. Nephrol. 2023, 19, 509. 37165096 10.1038/s41581-023-00720-1 PMC10171171

[40] L. Fugger , L. T. Jensen , J. Rossjohn , Cell 2020, 181, 63. 32243797 10.1016/j.cell.2020.03.007

[41] P. S. Serra , Nat. Biotechnol. 2019, 37, 238. 30804535 10.1038/s41587-019-0015-4

[42] X. Han , S. Huang , P. Xue , J. Fu , L. Liu , C. Zhang , L. Yang , L. Xia , L. Sun , S. K. Huang , T. Zhou , Sci. Adv. 2019, 5, eaax9230. 31844669 10.1126/sciadv.aax9230 PMC6905863

[43] P. Jorquera‐Cordero , L. J. Lara , T. Cruz , A. Schomann , T. G. van Hofslot , P. M. D. M. de Carvalho , L. Guedes , R. I. Creemers , A. B. Koning , R. F. Chan , J. de Araujo , Pharmaceutics 2022, 14, 1068. 35631654 10.3390/pharmaceutics14051068 PMC9143936

[44] L. G. Rikkert , R. Nieuwland , L. W. M. N. Terstappen , F. A. W. Coumans , J. Extracell. Vesicles 2019, 8, 1555419. 30651939 10.1080/20013078.2018.1555419 PMC6327933

[45] A. N. Oshchepkova , V. Matveeva , L. Artemyeva , K. Morozova , E. Kiseleva , M. Zenkova , V. Vlassov , Micromachines 2019, 10, 750. 31683842 10.3390/mi10110750 PMC6915531

[46] M. C. Ribeiro , V. L. R. Corrêa , F. K. L. da Silva , J. R. de Oliveira Neto , A. A. Casas , L. B. de Menezes , A. C. Amaral , Int. J. Biol. Macromol. 2018, 119, 32. 30031823 10.1016/j.ijbiomac.2018.07.119

[47] H. Kim , S. Y. Wang , G. Kwak , Y. Yang , I. C. Kwon , S. H. Kim , Adv. Sci. 2019, 6, 1900513. 10.1002/advs.201900513 PMC6794619 31637157

[48] G. Wu , J. Zhang , Q. Zhao , W. Zhuang , J. Ding , C. Zhang , H. Gao , D. W. Pang , K. Pu , H. Y. Xie , Angew. Chem., Int. Ed. 2020, 59, 4068. 10.1002/anie.201913700 31854064

[49] V. Le , J. Suh , J. J. Choi , G. T. Park , J. W. Lee , G. Shim , Y. K. Oh , ACS Nano 2019, 13, 7442. 31180642 10.1021/acsnano.9b02071

[50] J. Park , Q. V. Le , Y. Wu , J. Lee , Y. K. Oh , Adv. Mater. 2023, 35, 2202670. 10.1002/adma.202202670 36208089

[51] H. F. Alonso , S. Lemoine , C. Sedlik , E. Bottasso , I. Péguillet , V. Prémel , J. Denizeau , M. Salou , A. Darbois , N. G. Núñez , B. Salomon , D. Gross , E. Piaggio , O. Lantz , Nat. Commun. 2018, 9, 2113. 29844317 10.1038/s41467-018-04524-x PMC5974295

[52] K. Kirschbaum , J. K. Sonner , M. W. Zeller , K. Deumelandt , J. Bode , R. Sharma , T. Krüwel , M. Fischer , A. Hoffmann , M. Costa da Silva , M. U. Muckenthaler , W. Wick , B. Tews , J. W. Chen , S. Heiland , M. Bendszus , M. Platten , M. O. Breckwoldt , Proc. Natl. Acad. Sci. USA 2016, 113, 13227. 27799546 10.1073/pnas.1609397113 PMC5135308

[53] H. Guldner , S. M. Golomb , Q. Wang , E. Wang , S. Zhang , STAR Protoc. 2021, 2, 100537. 34036283 10.1016/j.xpro.2021.100537 PMC8138863

[54] L. d. O. Coser , M. T. Comelis , D. E. d. C. Matoso , L. P. Cartarozzi , A. L. R. d. Oliveira , Neuroglia 2024, 5, 129.

[55] P. C. D. Giles , N. M. Wilkinson , J. M. Washnock‐Schmid , B. M. Segal , J. Clin. Investig. 2018, 128, 5322. 30226829 10.1172/JCI123708 PMC6264723

[56] L. K. W. Giladi , H. Li , D. Dörr , C. Medaglia , F. Paul , A. Shemer , S. Jung , S. Yona , M. Mack , A. Leutz , I. Amit , A. Mildner , Nat. Immunol. 2020, 21, 525. 32313246 10.1038/s41590-020-0661-1

[57] A. P. Mrdjen , F. J. Hartmann , B. Schreiner , S. G. Utz , B. P. Leung , I. Lelios , F. L. Heppner , J. Kipnis , D. Merkler , M. Greter , B. Becher , Immunity 2018, 48, 380. 29426702 10.1016/j.immuni.2018.01.011

[58] M. Lam , K. Takeo , R. G. Almeida , M. H. Cooper , K. Wu , M. Iyer , H. Kantarci , J. B. Zuchero , Nat. Commun. 2022, 13, 5583. 36151203 10.1038/s41467-022-33200-4 PMC9508103

