# Supplementary material for: Anti‐Inflammatory Macrophage‐Derived Exosomes Modified With Self‐Antigen Peptides for Treatment of Experimental Autoimmune Encephalomyelitis
Source: Adv Sci (Weinh). 2025 Feb 12;12(13):2415265. doi: 10.1002/advs.202415265 (PMC11967809; doi:10.1002/advs.202415265)
Supplement: Supplementary file 1 — Supporting Information [file ADVS-12-2415265-s001.docx]

**Supplementary Video Information**

**
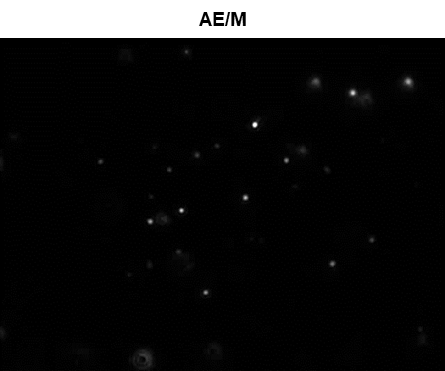
**

**Supplementary Video 1.** Nanoparticle tracking analysis of AE/M showing the movement of AE/M under Brownian motion.

**
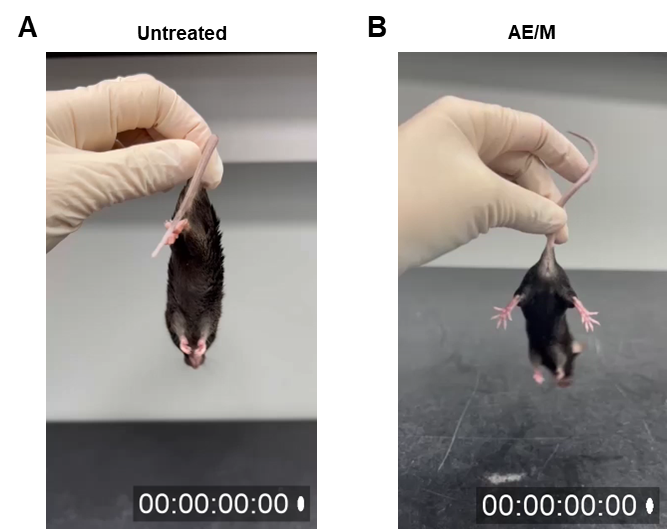
**

**Supplementary Video 2.** Motor coordination in mice treated with AE/M. A) Video recording of untreated EAE mice. B) Video recording of mice treated with AE/M.

**Supplementary Figures**


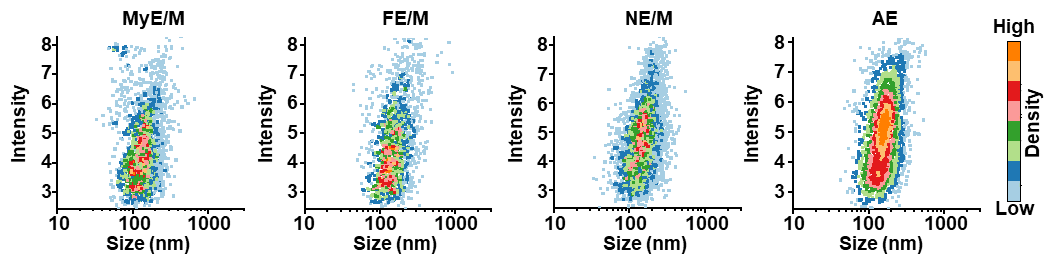


**Figure S1.** Size distribution of various exosomes. Size distribution was measured by nanoparticle tracking analysis.


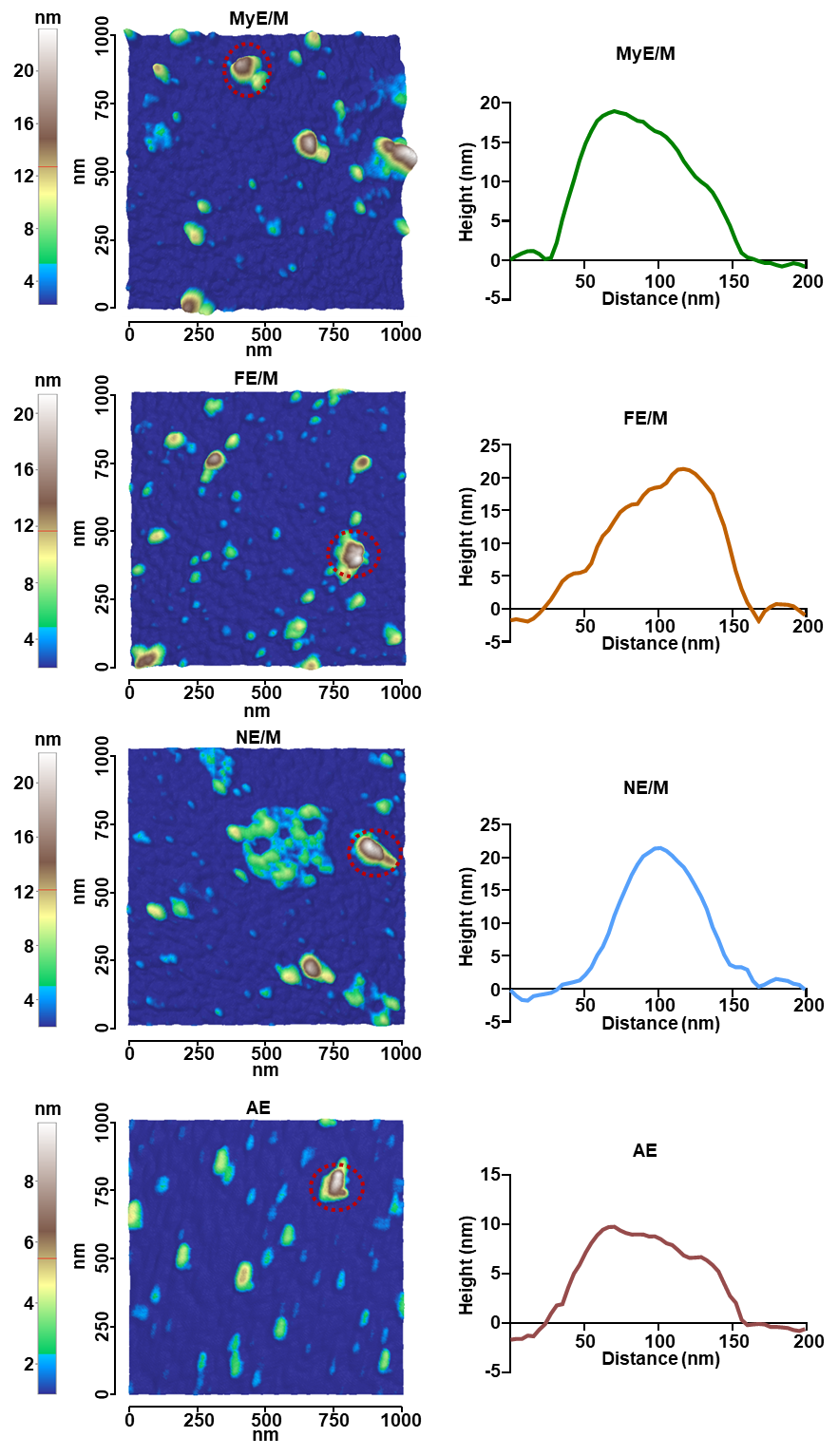


**Figure S2.** AFM images of various exosomes. The dimension of the exosomes was highlighted in the left panel and heights was shown in the right panel.


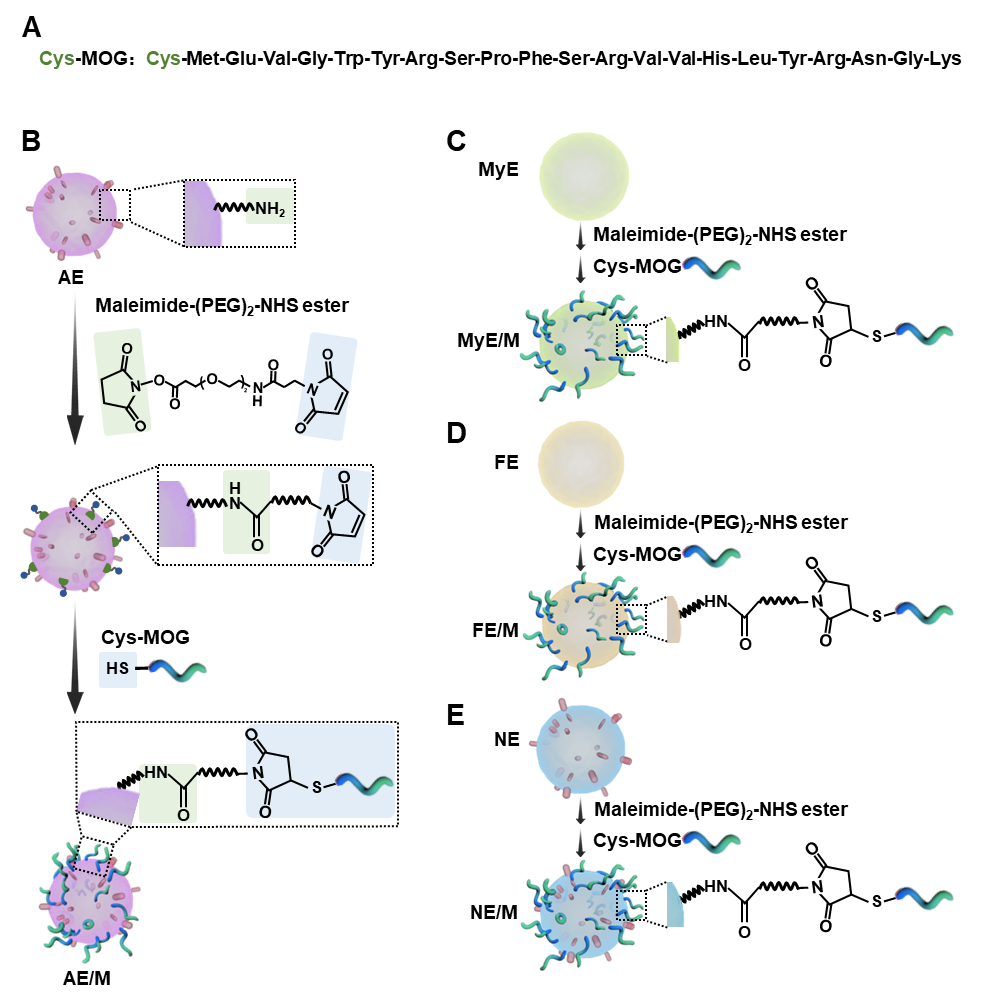


**Figure S3.** The conjugation of MOG peptides to various exosomes. A) The sequence of cysteine-modified MOG peptide. B) Conjugation procedures of MOG onto exosomes. Firstly, maleimide-(PEG)_2_-NHS ester was mixed with exosomes where NHS ester reacted with amine groups on exosomes. Next, cysteine-modified MOG peptide was conjugated to exosomes via the thiol group and maleimide moiety. C-E) The modification of MyE, FE, and NE with cysteine-modified MOG peptide: MyE (C), FE (D), and NE (E).


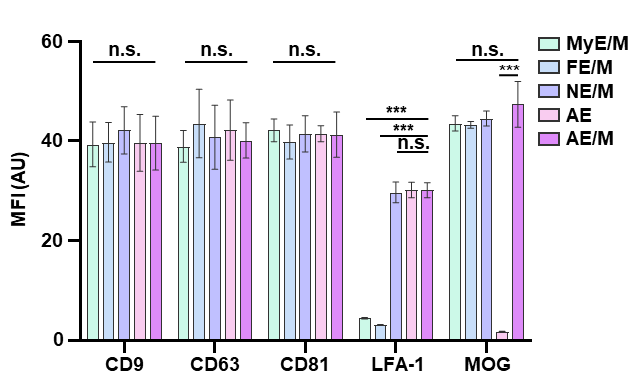


**Figure S4.** Quantitative analysis of exosome surface markers. The mean fluorescence intensity of exosome surface markers was determined based on fluorescence images obtained through confocal microscopy (*n* = 5). (Data are presented as the mean ± SD. n.s., not significant; ****p* < 0.001).


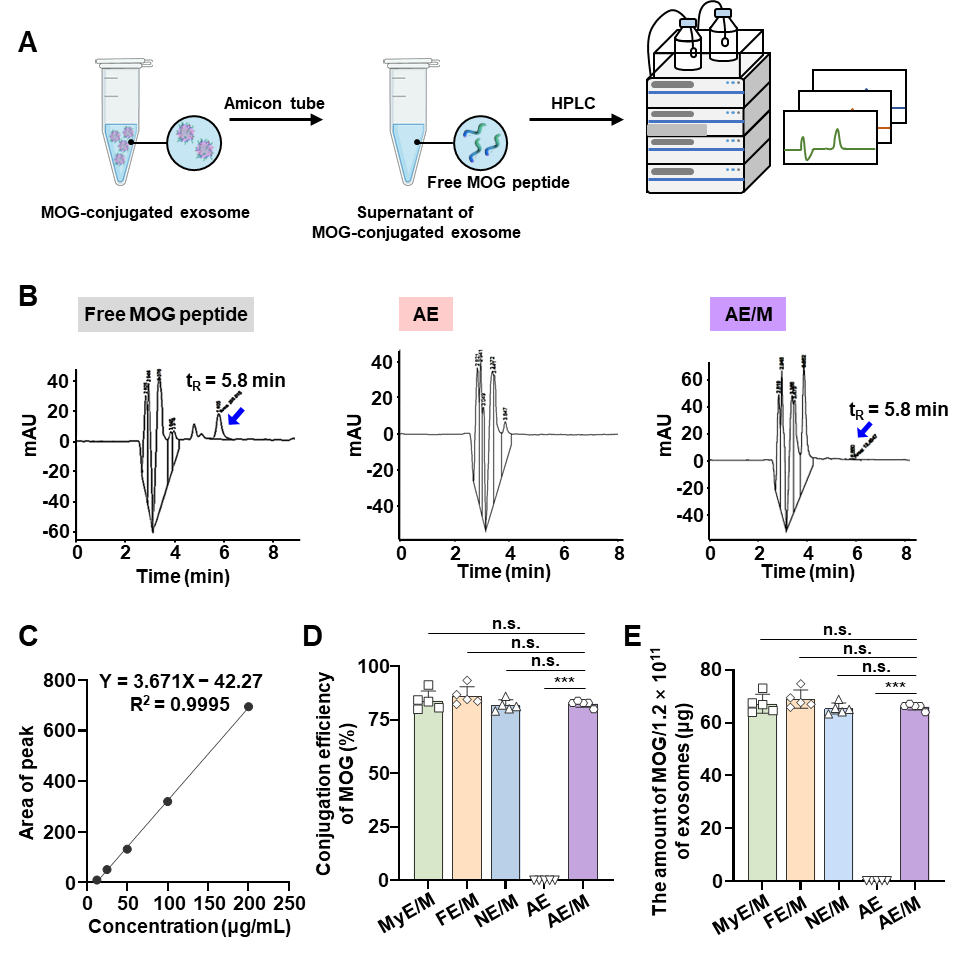


**Figure S5.** Quantification of MOG conjugation. A) The flow of the MOG quantification analysis on exosomes is illustrated. B) The HPLC profile of MOG peptides shows the retention time of the MOG peptides in min. C) The standard curve of MOG peptides is presented for quantification purposes. D) The conjugation efficiency of MOG peptides to exosomes is shown (*n* = 5). E) The amount of MOG peptides per 1.2 × 10¹¹ exosomes is quantified (*n* = 5). (Data are presented as the mean ± SD. n.s., not significant; ****p* < 0.001).


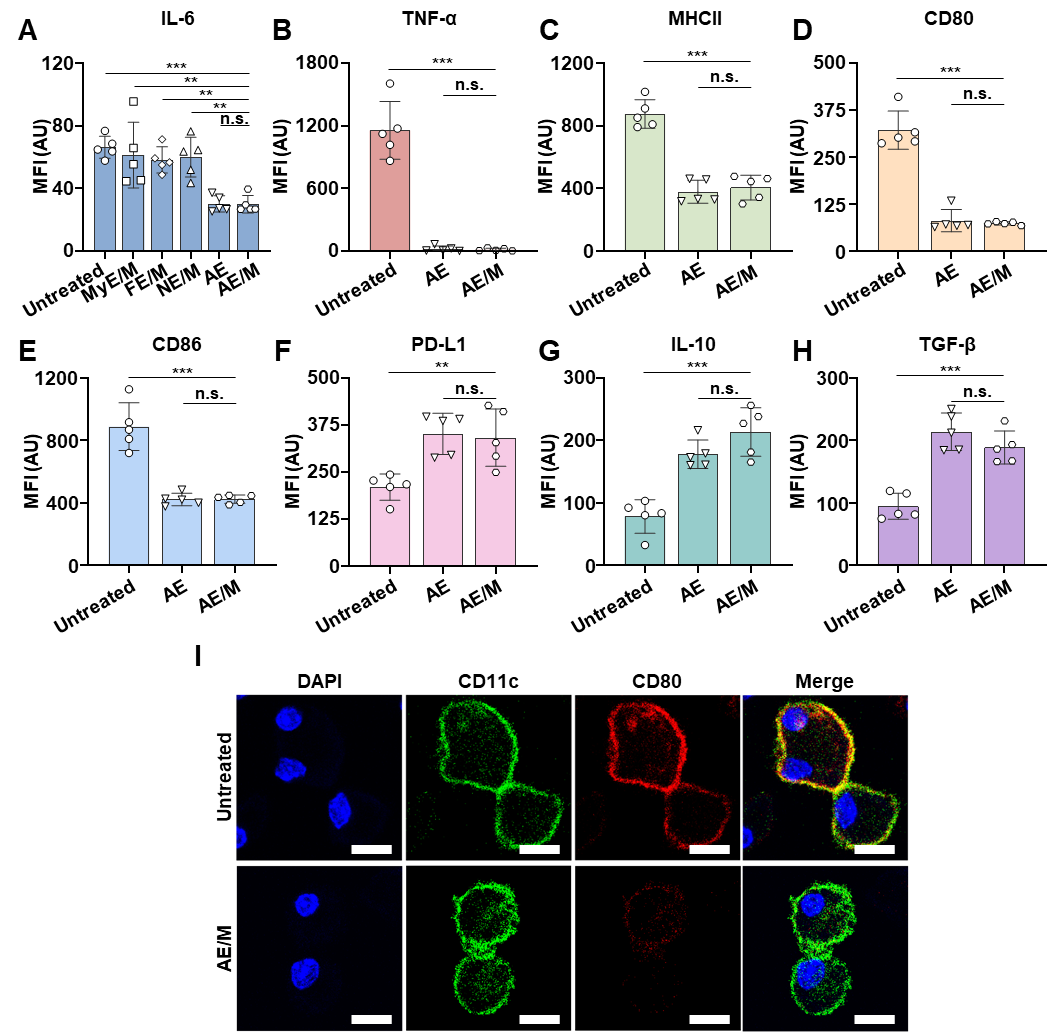


**Figure S6.** The expression of inflammation markers in DC after treatment of exosomes. A) The expression of IL-6 in DC after the treatment of various formulations (*n* = 5). B-E) The expression of pro-inflammatory markers after the treatment of AE and AE/M: TNF-*α* (B); MHCII (C); CD80 (D); CD86 (E) (*n* = 5). F-H) The expression of anti-inflammatory markers after the treatment of AE and AE/M: PD-L1 (F); IL-10 (G); TGF-*β* (H) (*n* = 5). I) The expression of CD80 in DC after AE/M treatment visualized through confocal microscopy. Scale bar: 10 μm. (Data are presented as the mean ± SD. n.s., not significant; ***p* < 0.01; ****p* < 0.001).


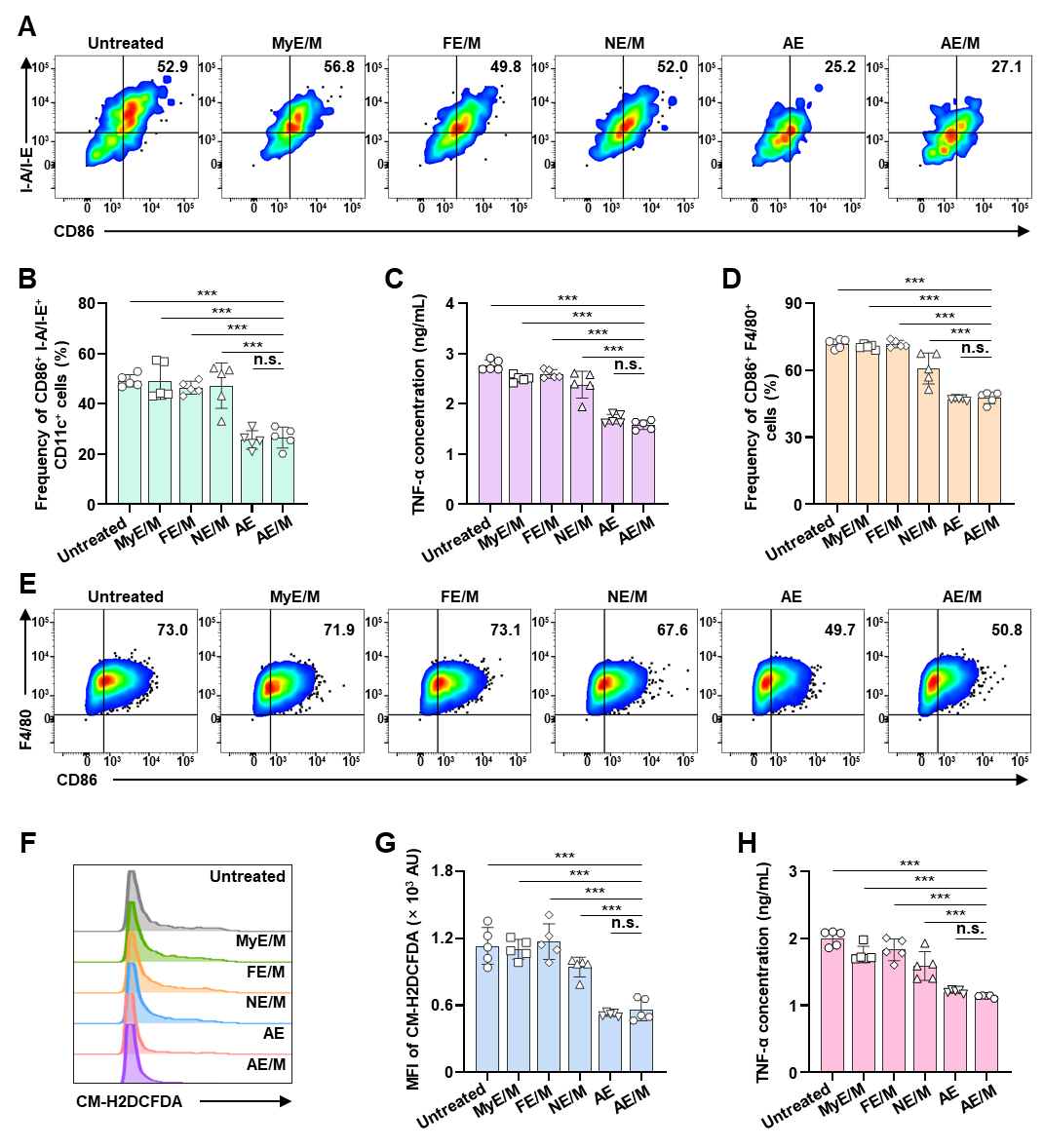


**Figure S7.** Effect of exosomes on BMDC and BMDM. A) Plots show CD86^+^ BMDC after treatment with various exosomes. B) The percentage of CD86^+^ BMDC was assessed by flow cytometry (*n* = 5). C) The level of TNF-*α* in the BMDC culture medium was quantified using TNF-*α* ELISA kit (*n* = 5). D) The percentage of CD86^+^ BMDM was determined by flow cytometry (*n* = 5). E) Plots show CD86^+^ BMDM after treatment with exosomes. F) Flow cytometric analysis was performed for CM-H2DCFDA staining to measure intracellular ROS in BMDM. G) CM-H2DCFDA fluorescence intensity was quantified (*n* = 5). H) The level of TNF-*α* in the BMDM culture medium was quantified using TNF-*α* ELISA kit (*n* = 5). (Data are presented as the mean ± SD. n.s., not significant; ****p* < 0.001).


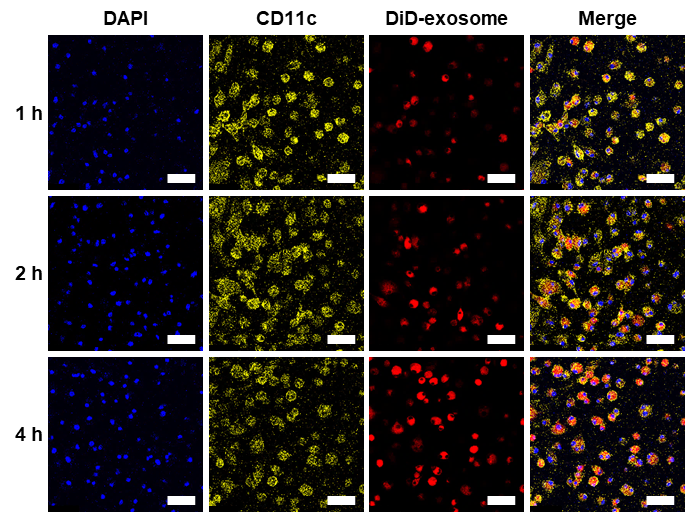


**Figure S8.** Uptake of AE/M by DC. Cells were treated with DiD-labeled AE/M and observed at different time points by confocal microscopy. Scale bar: 50 μm.


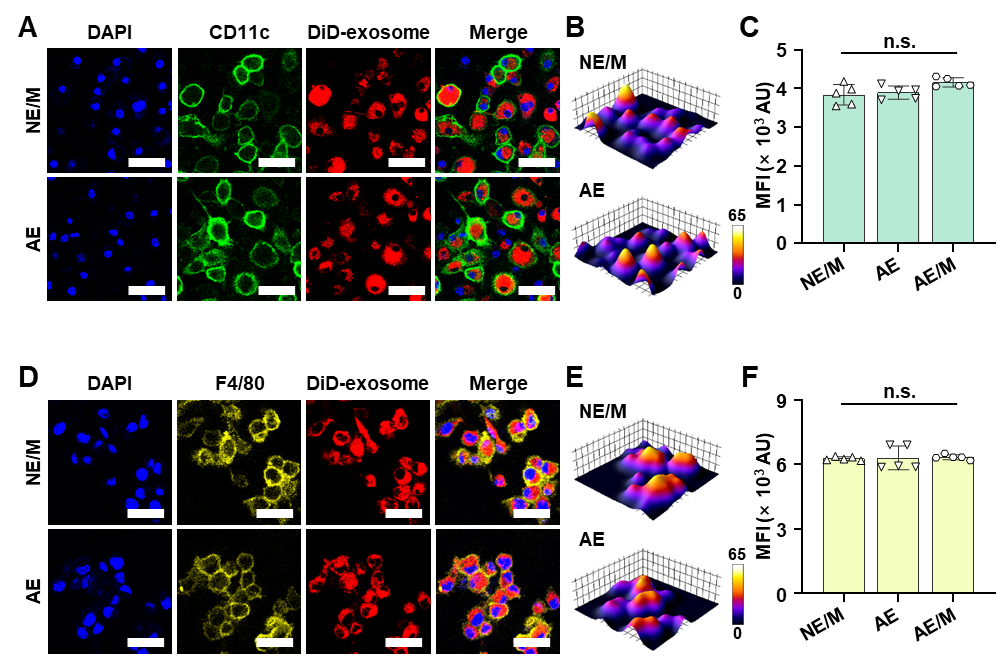


**Figure S9.** Uptake of macrophage-derived exosomes by DC and macrophages. A) Uptake of macrophage-derived exosomes by DC was visualized by confocal microscopy. Scale bar: 40 μm. B) 3D surface plot of DiD-exosome obtained from confocal images shown in (A). C) Mean fluorescence intensity of internalized macrophage-derived exosomes by DC (*n* = 5). D) Uptake of macrophage-derived exosomes by macrophages was visualized by confocal microscopy. Scale bar: 20 μm. E) 3D surface plot of DiD-exosome obtained from confocal images shown in (D). F) Mean fluorescence intensity of internalized macrophage-derived exosomes by macrophages (*n* = 5). (Data are presented as the mean ± SD. n.s., not significant).


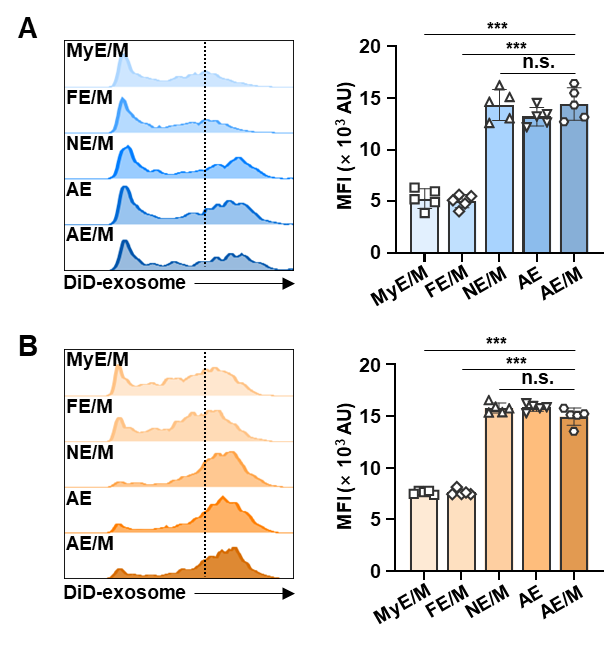


**Figure S10.** Cellular uptake of various exosomes. A-B) The cellular uptake of each exosome group in splenic dendritic cells (A) and macrophages (B) was assessed by flow cytometry (*n* = 5). Splenocytes from EAE mice were treated with DiD-labeled exosome nanoparticles, followed by flow cytometry analysis. (Data are presented as the mean ± SD. n.s., not significant; ****p* < 0.001).


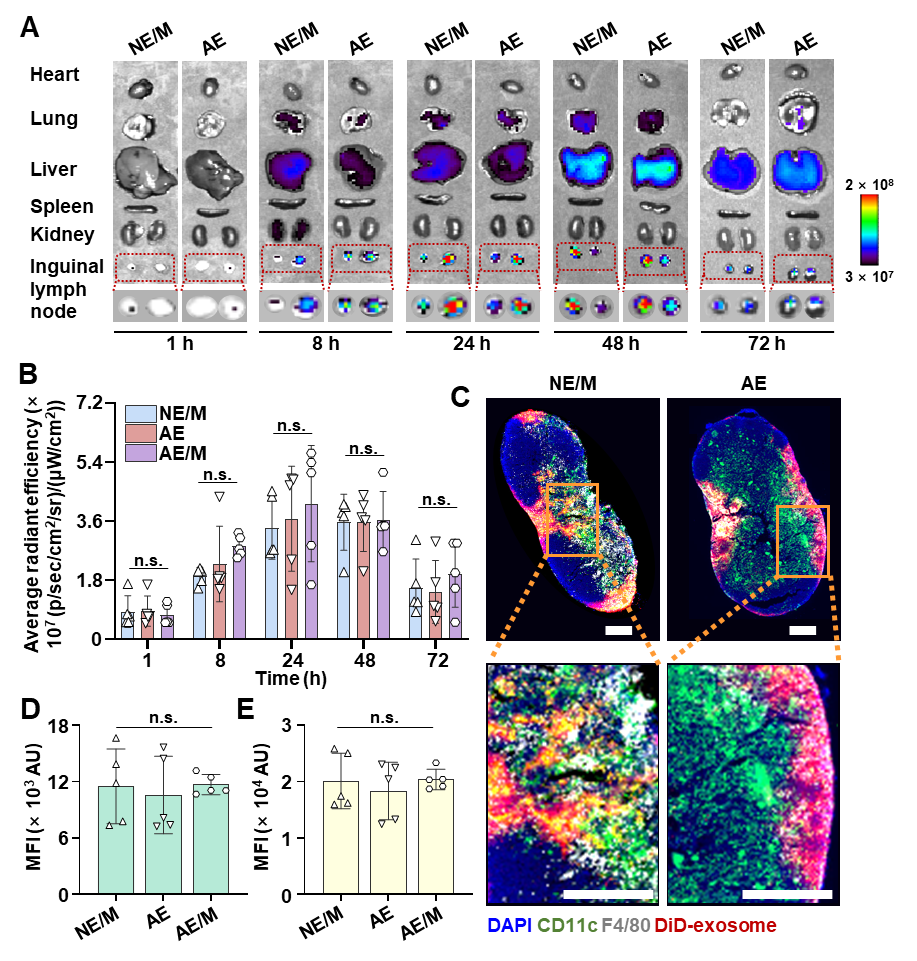


**Figure S11.** Biodistribution of macrophage-derived exosomes. A) Fluorescence images showing the biodistribution of macrophage-derived exosomes were obtained using the IVIS instrument. DiD-labeled exosome formulations were administered subcutaneously, and *in vivo* fluorescence imaging was performed at different time points using the IVIS instrument. B) The fluorescence intensity of macrophage-derived exosomes in the inguinal lymph nodes was measured at different time points (*n* = 5). C) Immunofluorescence images show macrophage-derived exosomes in lymph nodes 24 h after subcutaneous administration. Immunostaining was performed on tissue sections using DAPI (blue), anti-CD11c antibody (green), and anti-F4/80 antibody (gray). The scale bars indicate 0.25 mm for the upper and lower images. D) Mean fluorescence intensity of macrophage-derived exosomes taken up by DC (*n* = 5). E) Mean fluorescence intensity of macrophage-derived exosomes internalized by macrophages (*n* = 5). (Data are presented as the mean ± SD. n.s., not significant).

**
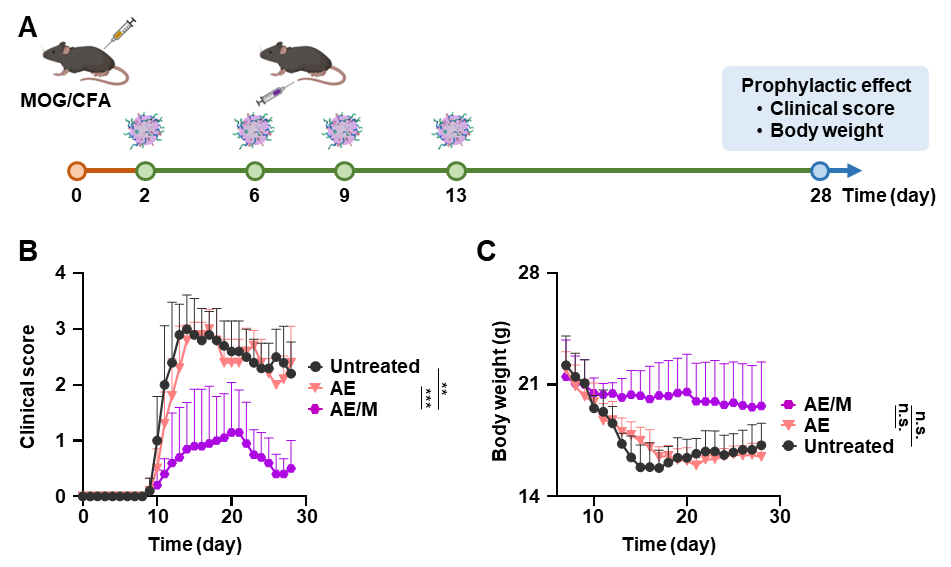
**

**Figure S12.** *In vivo* prophylactic effect of intravenously administered exosomes in EAE mice. A) A schematic illustration shows the schedule of the *in vivo* study. Two days after EAE induction (prior to EAE onset), exosomes were intravenously injected on days 2, 6, 9, and 13. Clinical scores and body weights were recorded throughout the study. B) The clinical scores of mice treated with different exosomes were measured (*n* = 5). C) The body weights of mice in each group were recorded (*n* = 5). (Data are presented as the mean ± SD. n.s., not significant; ***p* < 0.01; ****p* < 0.001).

**
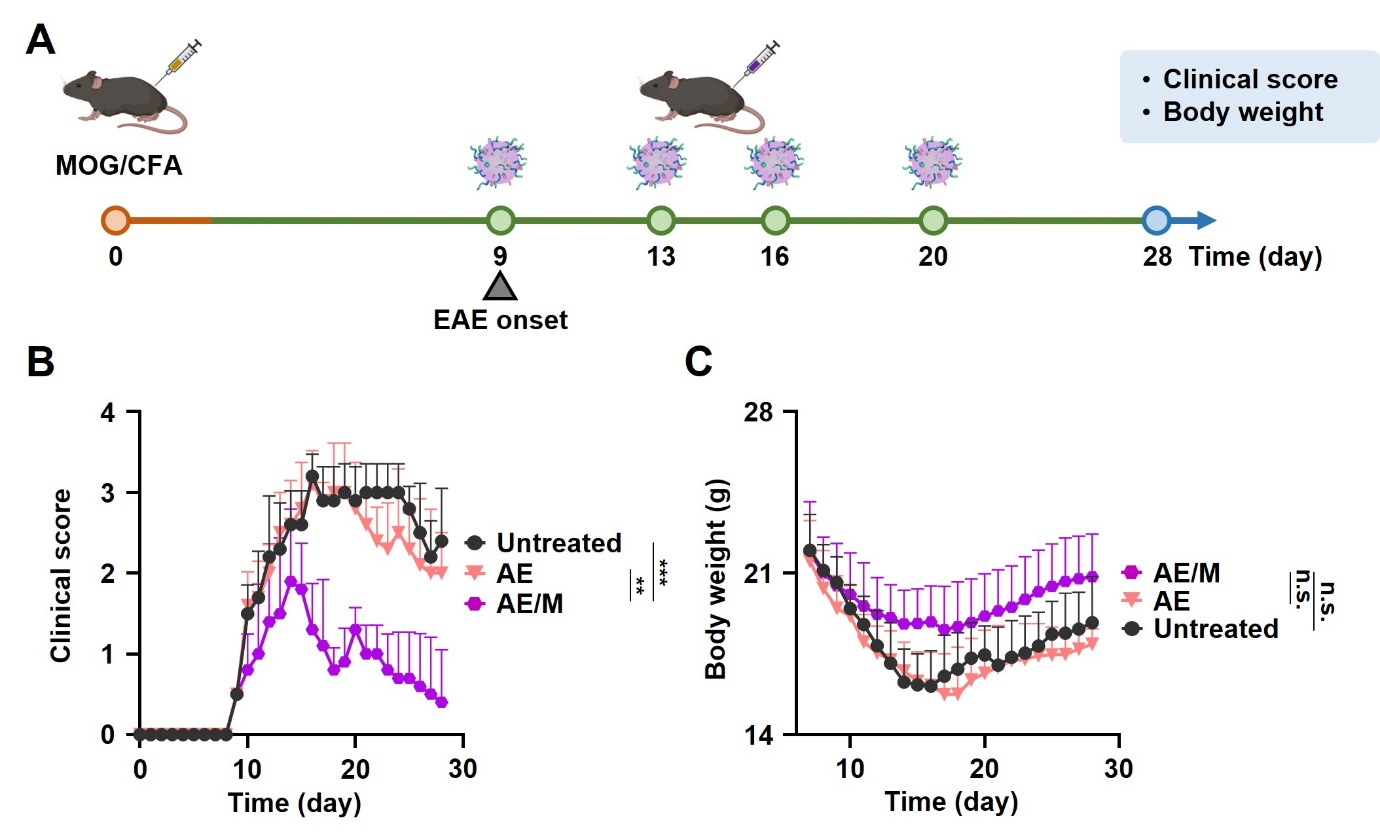
**

**Figure S13.** *In vivo* prophylactic effect after symptom onset in EAE mice. A) The experimental schedule of the *in vivo* study is outlined. Treatment was initiated at the onset of EAE (on day 9 post-EAE induction). AE or AE/M were administered via subcutaneous injection on days 9, 13, 16, and 20. Clinical scores and body weights were recorded throughout the study. B) The clinical scores of mice were measured (*n* = 5). C) The body weights of mice in each group were recorded (*n* = 5). (Data are presented as the mean ± SD. n.s., not significant; ***p* < 0.01; ****p* < 0.001).

**
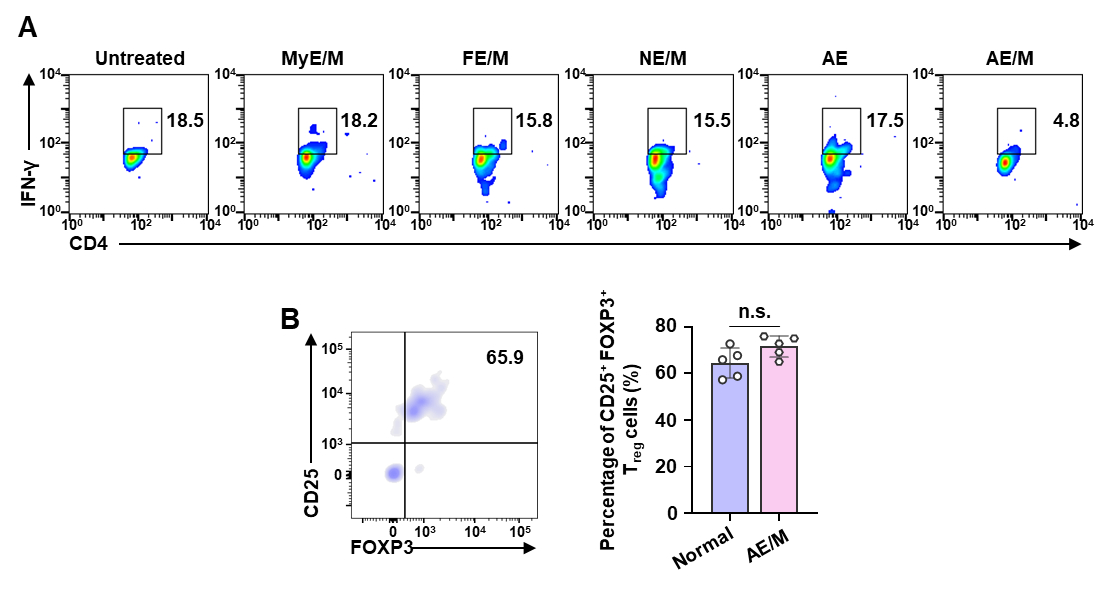
**

**Figure S14.** Immune cell profiling in the CNS. A) Plots showing IFN-*γ*^+^ CD4^+^ T cells in spinal cords analyzed by flow cytometry. B) The frequency of T_reg_ cells in the CNS of normal mice and EAE mice treated with AE/M was assessed (*n* = 5). The plot shows T_reg_ cells in the CNS of normal mice. (Data are presented as the mean ± SD. n.s., not significant).

**
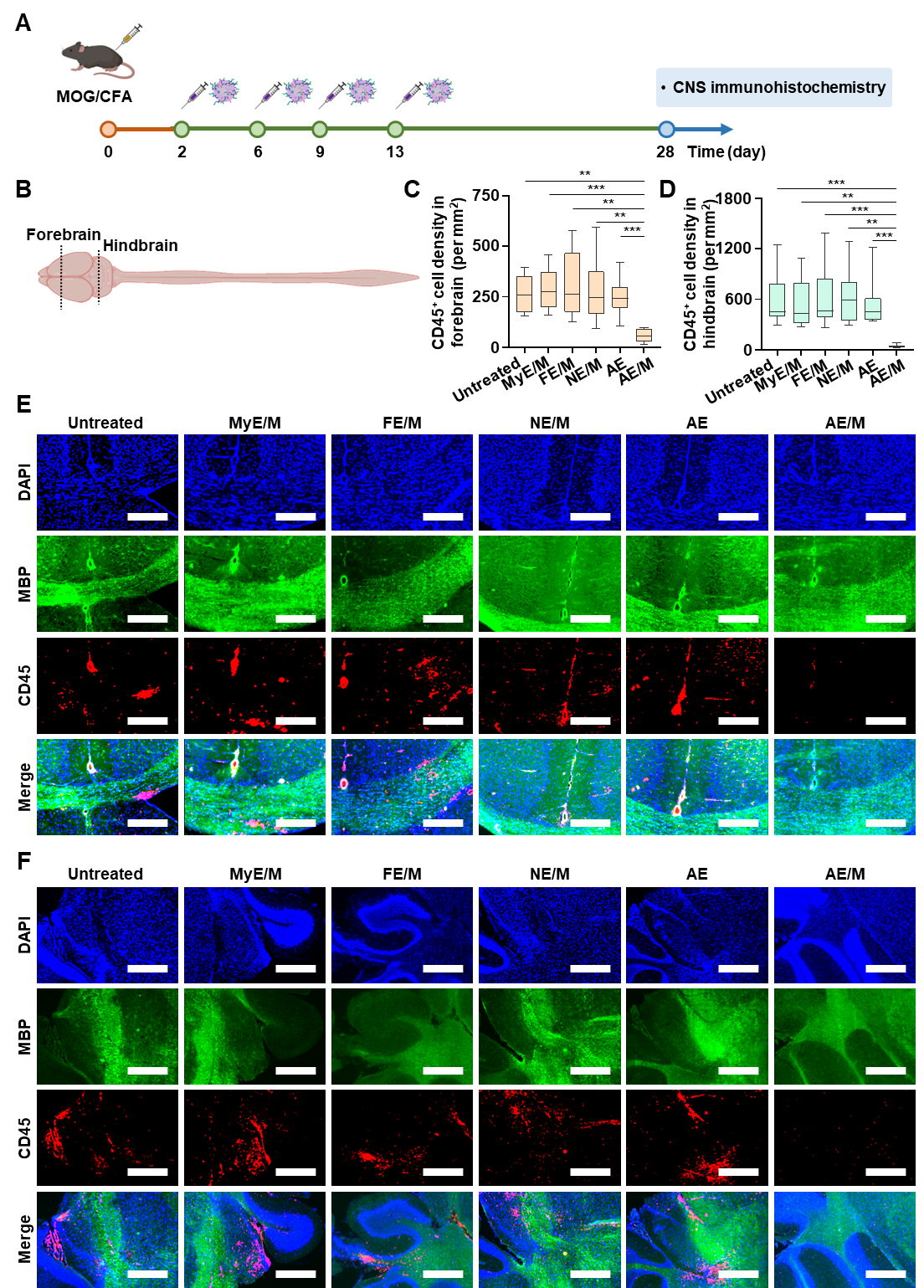
**

**Figure S15.** Infiltration of leukocytes to the forebrain and hindbrain. A) A schematic illustration depicts the schedule of the *in vivo* study. Subcutaneous administration of various exosome formulations began 2 days after EAE model induction. At the end of the study, brains were harvested to prepare tissue sections. B) Tissue section sites in the forebrain and hindbrain were selected for the immunohistochemistry study. C-D) The density of CD45^+^ cells in different brain sections were analyzed using the Vectra tissue analyzer in the forebrain (C) and hindbrain (D) (*n* = 10). E-F) Immunofluorescence images of leukocytes in brain tissues were captured using the THUNDER imaging system, showing the forebrain (E) with a scale bar of 0.3 mm and the hindbrain (F) with a scale bar of 0.5 mm. Immunostaining was performed using DAPI (blue), anti-MBP antibody (green), and anti-CD45 antibody (red). (Data are presented as the mean ± SD. ***p* < 0.01; ****p* < 0.001).

**
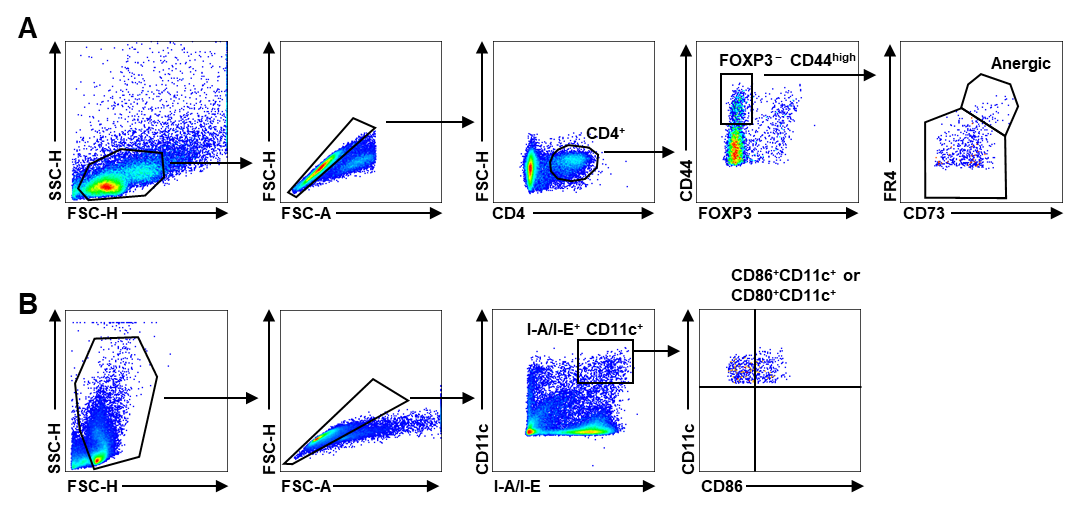
**

**Figure S16.** Gating strategy for immunophenotyping in spleens. A) CD4^+^ T cells were first gated, followed by the gating of FOXP3^−^ CD44^high^ cells. Anergic T cells were identified as FR4^high^ CD73^high^ cells within FOXP3^−^ CD44^high^ CD4^+^ T cells. B) Dendritic cells were gated as I-A/I-E^+^ CD11c^+^ cells, and the expression of CD86 and CD80 was further analyzed.

**
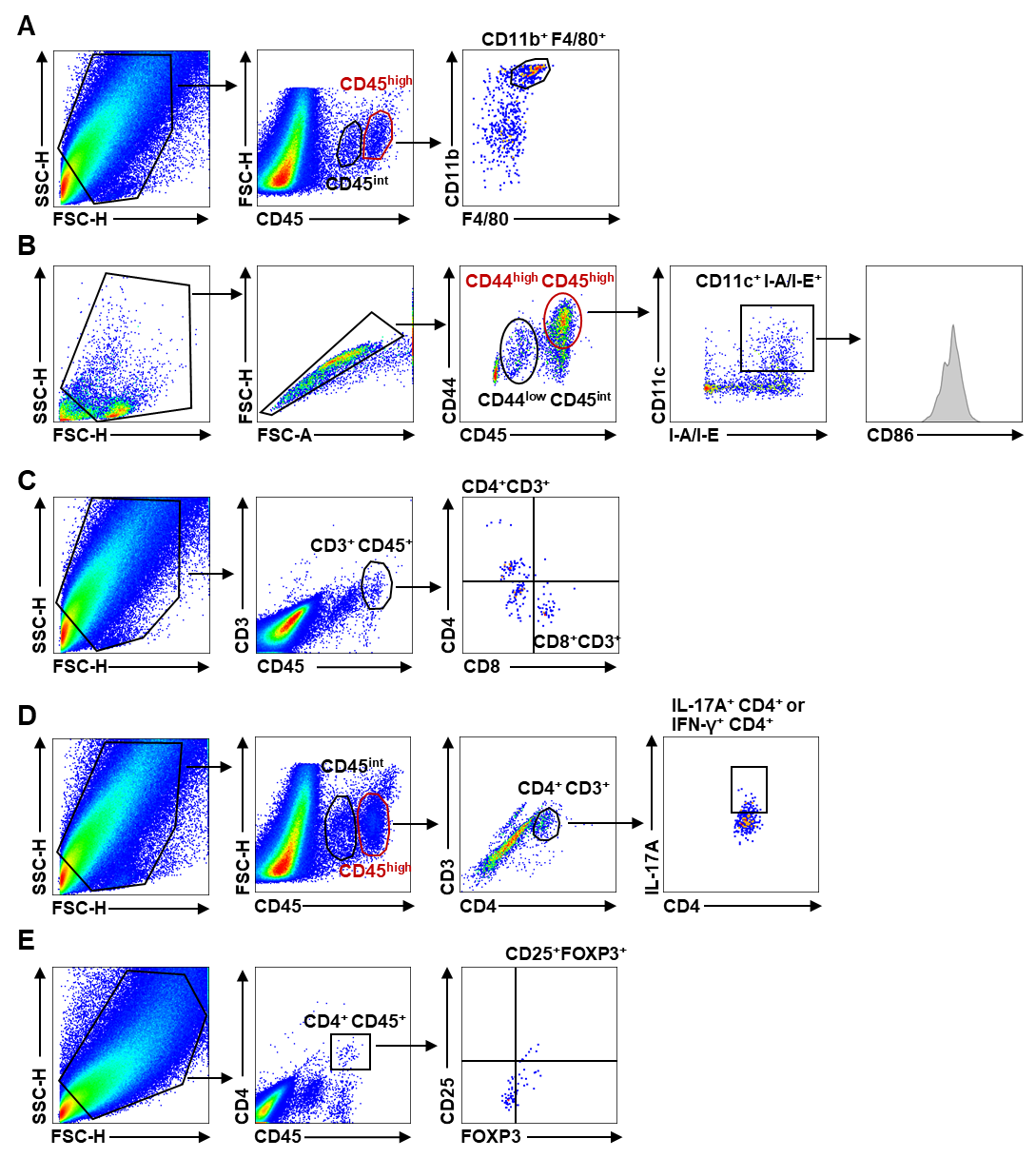
**

**Figure S17.** Gating strategy for immune cell profiling in the CNS. A) Macrophage gating strategy. Leukocytes were identified as CD45^high^ cells and macrophages were further gated as CD11b^+^ F4/80^+^ cells. B) DC gating strategy. Cells were initially gated as CD44^high^ CD45^high^, with DC identified by further gating of CD11c^+^ I-A/I-E^+^ cells. The expression levels of CD86 and CD80 were then analyzed in these DC. C) T cell gating strategy. T cells were first identified as CD3^+^ CD45^+^ cells, with further distinction into CD4^+^ CD3^+^ T cells and CD8^+^ CD3^+^ T cells. D) Gating strategy for analysis of intracellular cytokines in CD4^+^ T cells: CD45^high^ cells were gated as leukocytes, with CD4^+^ T cells further identified by CD3 and CD4 markers. Intracellular cytokines including IL-17A and IFN-*γ* were evaluated in these CD4^+^ T cells. E) T_reg_ cell gating strategy in CNS. CD4^+^ T cells were first identified as CD4^+^ CD45^+^ cells, with further gating to identify T_reg_ cells as CD25^+^ FOXP3^+^ CD4^+^ T cells.
